# Spatial variation of premarital HIV testing and its associated factors among married women in Ethiopia: Multilevel and spatial analysis using 2016 demographic and health survey data

**DOI:** 10.1371/journal.pone.0293227

**Published:** 2023-11-30

**Authors:** Werkneh Melkie Tilahun, Tigabu Kidie Tesfie

**Affiliations:** 1 Department of Public Health, College of Medicine and Health Sciences, Debre Markos University, Debre Markos, Ethiopia; 2 Department of Epidemiology and Biostatistics, Institute of Public Health, College of Medicine and Health Sciences, University of Gondar, Gondar, Ethiopia; Madda Walabu University, ETHIOPIA

## Abstract

**Background:**

Africa is the most severely affected area, accounting for more than two-thirds of the people living with HIV. In sub-Saharan Africa, more than 85% of new HIV-infected adolescents and 63% of all new HIV infections are accounted for by women. Ethiopia has achieved a 50% incidence rate reduction. However, mortality rate reduction is slow, as the estimated prevalence in 2021 is 0.8%. In sub-Saharan Africa, heterosexual transmission accounts for the majority of HIV infections, and women account for 58% of people living with HIV. Most of these transmissions took place during marriage. Thus, this study aimed to explore the spatial variation of premarital HIV testing across regions of Ethiopia and identify associated factors.

**Methods:**

A cross-sectional study design was employed. A total of 10223 weighted samples were taken from individual datasets of the 2016 Ethiopian Demographic and Health Survey. STATA version 14 and ArcGIS version 10.8 software’s were used for analysis. A multilevel mixed-effect generalized linear model was fitted, and an adjusted prevalence Ratio with a 95% CI and p-value < 0.05 was used to declare significantly associated factors. Multilevel models were compared using information criteria and log-likelihood. Descriptive and spatial regression analyses (geographical weighted regression and ordinary least squares analysis) were conducted. Models were compared using AICc and adjusted R-squared. The local coefficients of spatial explanatory variables were mapped.

**Results:**

In spatial regression analysis, secondary and above education level, richer and above wealth quintile, household media exposure, big problem of distance to health facility, having high risky sexual behaviour and knowing the place of HIV testing were significant explanatory variables for spatial variation of premarital HIV testing among married women. While in the multilevel analysis, age, education level, religion, household media exposure, wealth index, khat chewing, previous history of HIV testing,age at first sex, HIV related knowledge, HIV related stigma, distance to health facility, and community level media exposure were associated with premarital HIV testing among married women.

**Conclusions and recommendation:**

Premarital HIV testing had a significant spatial variation across regions of Ethiopia. A statistically significant clustering of premarital HIV testing was observed at Addis Ababa, Dire Dawa, North Tigray and some parts of Afar and Amhara regions. Therefore area based prevention and interventional strategies are required at cold spot areas to halt the role of heterosexual transmission in HIV burden. Moreover, the considering the spatial explanatory variables effect in implementations of these strategies rather than random provision of service would make regional health care delivery systems more cost-effective.

## Introduction

Since the start of the epidemic, there have been 84.2 million new HIV infections worldwide and 40.1 million deaths due to the virus [1]. As of the end of 2021, 38.4 million individuals were living with the virus. A global estimate of 0.7% of adults between the ages of 15 and 49 have HIV, despite the fact that the severity of the epidemic differs between different countries and regions [1]. The African Region remains the most severely affected area, accounting for more than two-thirds of the people living with HIV worldwide [1–3]. Around 4900 teenage girls between the ages of 15 and 24 contract HIV every week throughout the world. And by 2021, women and girls would have been responsible for 63% of all new HIV infections in this region due to the fact that more than 85% of new HIV infections among 15 to 19-year-olds in sub-Saharan Africa occur in females, putting them at a twofold greater risk than their male counterparts [3].

HIV prevalence in Ethiopia increased alarmingly from 1.7 to 3% between 1990 and 1995 and was predicted to be 0.8% in 2021 [4]. Since 1995, 6.3% and 0.4% annual incidence and mortality reduction rates have been recorded, respectively. As a result, Ethiopia has met the Millennium Development Goals’ aim of a 50% reduction in the incidence rate of HIV/AIDS. However, the rate of mortality reduction has been comparatively slow [5].

The virus has been linked to various ways of transmission. Women make up 58% of HIV-positive individuals in sub-Saharan Africa, where heterosexual transmission is the primary cause of HIV infections [2]. For both men and women, marriage or cohabitation is where the majority of this heterosexual HIV transmission occurs [6]. Pre-marital HIV screening is the process of detecting a couple’s (prospective or intending) HIV status in order to reduce the possibility of HIV transmission to the kids. Marriage is not permitted when there is a sero-discordance in the majority of African nations, including Ethiopia. As a result, more couples are freely asking for health examinations before getting married. Following the results and related clinical recommendations, prospective spouses are not required to follow them if they choose not to. Thus, couples who test together could have both positive and negative challenges to their relationships [7].

Typically, sexual activity begins prior to marriage, and many women are HIV-positive when they get married in sub-Saharan Africa. Even though it’s possible that a sizable percentage of males could have HIV before getting married, this proportion is smaller than it is for women [8]. As a crucial first step toward HIV care and treatment, HIV testing and counseling are therefore essential for preventing HIV transmission and will continue to be an integral aspect of preventive efforts [9–11]. Besides, achievements in HIV prevention in sub-Saharan Africa could change the course of the disease’s worldwide impact, given that it is still difficult to provide HIV prevention, treatment, and initiatives to stop sexual HIV transmission [2].

Despite the fact that couples voluntary counseling testing (CVCT) has long been advocated for HIV prevention by promoting safer sexual behavior and increasing disclosure between sexual partners [11, 12], few couples have been reached [13, 14]. Therefore, it’s critical to encourage couples to regularly seek HIV counseling and testing, especially if they intend to engage in unprotected sex [15].

Unmarried cohabiting couples have a high rate of HIV infection [15]. One method of preventing sexual transmission of HIV to partners is premarital HIV testing, which has significantly decreased the sero-positivity rate among mothers [11, 16, 17]. This early case identification and counseling is regarded as one of the most efficient strategies in the Prevention of Maternal to Child Transmission (PMTCT) program because it gives the HIV-positive couple the chance to make a wise choice before entering into a marriage and subsequently aids in anticipating how they will manage themselves and their future children [11, 16–18].

A number of biological, social, behavioral, cultural, economic, and structural factors have combined in SSA to cause a disproportionally higher risk of HIV infection among women relative to their male counterparts [19]. Previous literature revealed that 20 and above years of age [20–24], urban by residence [20, 21, 25–27], primary and above educational level [20, 22, 25–29], protestant and other religion [21], access to media [25, 26, 29], middle and above household wealth index [20, 22, 25, 26, 29, 30], knowing the place of HIV testing [25, 26], being khat chewer [25, 26], alcohol drinking [25–27, 31], presence of HIV related stigma [24, 25], having HIV related knowledge [22, 24, 30], being currently employed [20, 28], visiting health facility in the last 12 months [24, 27], early initiation of sex [24], having recent sexual activity [20], having history of risky sexual behaviors [20, 21, 24], and community-level education [30] were positively associated with HIV testing. In contrast to the above mentioned studies wealth index [24], previous history of HIV testing [31], presence of HIV related stigma [21, 22, 30], having HIV related knowledge [21], and high community-level education [24] were negative associated with HIV testing.

Since 1985, Ethiopia has made an effort to combat the HIV/AIDS pandemic. Although the most recent version was released in 2002, the initial counseling and testing guidelines were first published in 1996. On top of the national VCT guidelines development, evidence-based practices have been implemented, which help to improve the effectiveness and accessibility of counseling and testing as well as their overall quality [11]. However, a relatively small number of couples have been receiving voluntary counseling and testing [32].

Different studies have been conducted in Ethiopia [25, 26, 33]. However, Two of them [25, 26] didn’t consider the hierarchical nature of the DHS data and used classical logistic regression to identify associated factors. Despite advances in statistical analysis techniques, inappropriate analysis remains a challenge in biomedical research [34]. One of them is failing to account for clustering effects and applying the classical regression model to clustered data, which generates extreme, biased, and invalid results and can generate misleading statistical inferences and false conclusions (unaccepted by the scientific community) [35–38]. This error is greatest when the outcome variable is more clustered, that is, when the ICC is ≥ 10% [35]. And the other reported the prevalence as 21.4% [33]. Even if it had considered the clustering effect, it identified factors associated with premarital HIV testing using logistic regression and reported the odds ratio. However, the odds ratio is not a good measure of association when the prevalence is greater than 10%, as the PR can be overestimated or underestimated by the odds ratio, which can be solved by applying robust poisson or log binomial regression [39, 40]. Accordingly, the previous studies had a methodological gap in their analysis, and appropriate statistical methods need to be applied in order to identify factors related to premarital HIV testing. Additionally, to examine the regional disparity of premarital HIV testing and spatial factors that influenced the geographic discrepancy across regions of Ethiopia, our study used geographically weighted regression. It is essential to pinpoint areas with low and high rates of premarital HIV testing as well as the underlying factors in order to develop context- and area-based interventions to fight the disease. In order to close this gap, the current study used a multilevel robust-Poisson and geographically weighted regression analysis to explore the spatial variation of premarital HIV testing across regions of Ethiopia and identify associated factors.

## Methods and materials

### Data sources, setting, population and sampling design

A cross-sectional study was conducted based on the 2016 Ethiopian Demographic and Health Survey (EDHS) dataset. It is a nationally representative household survey conducted at intervals of 5 years and provides a wide range of data on health and related characteristics like population and nutrition. Its samples are usually selected based on a stratified, two-stage cluster sampling technique. The sampling frame consists of 84,915 enumeration areas (EAs) based on the 2007 Ethiopia Population and Housing Census (PHC). Each EA covers, on average, 181 households. The survey was collected across the nine regional states and two city administrations of Ethiopia. First, each region was stratified into urban and rural areas, and then a total of 645 EAs (202 in urban areas and 443 in rural areas) were selected with a probability proportional to EA size based on the 2007 PHC. A fixed number of 28 households per cluster were selected with equal probability through systematic selection. Additional information about data collection, sampling, and questionnaires used in the surveys is explained in the 2016 EDHS report [41] **(S1 Fig)**.

The data was derived from (http://www.dhsprogram.com) up on an official online request and permission. The DHS has datasets for different populations such as men, women, children, births, and households.

### Participants

Our study was conducted based on women’s datasets (individual record files). A total weighted sample of 10,223reproductive-aged women who were married or lives with their partner were included in the study (**S1 Fig**).

### Variables of the study

#### Outcome variable

The main outcome of this study was the self-reported history of premarital HIV testing among reproductive-aged women who were married or lives with their partner (“yes” or “no”).

#### Independent variables

Factors related to premarital HIV testing were selected based on a literature review and include age, residence, region, education level, religion, access to media, wealth index, knowing the place of HIV testing, being a khat chewer and alcohol drinker, previous history of HIV testing, HIV-related stigma, HIV-related knowledge, employment, visiting a health facility, distance to a health facility, age at first sex, history of recent sexual activity, history of risky sexual behaviors, and community-level education and media exposure.

### Measurement and operational definitions

#### Household media exposure

Created by combining whether the participant’s mother reads a newspaper or magazine, listens to the radio, or watches television and coded as "yes" (if a woman had been exposed to at least one of these media) and "no" otherwise.

#### HIV-related knowledge

Created by combining six items. If a person responded that regular use of condoms and having one sexual partner can reduce the risk of HIV/AIDS, believed that a healthy-looking person can have HIV, and was aware that a person cannot get HIV through a mosquito bite, by sharing food with someone who has AIDS, or by witchcraft or supernatural means, the total sum was categorized as low (score ≤ 3), high (scores 4 and 5), or comprehensive (score 6) [21].

#### Religion

Recategorized as “Orthodox”, “Catholic”, “Protestant”, “Muslim” and “Other”.

#### HIV-related stigma

It was created by combining six items that reflect a negative attitude about HIV/AIDS. These were: (1) if a woman would not buy fresh vegetables from an HIV-positive vendor; (2) if a woman said HIV-positive children should not be allowed to attend school with children who do not have HIV; (3) If a respondent said a person was ashamed if a family member had HIV; (4) If a respondent said people hesitate to take an HIV test due to others reactions if positive; (5) If a respondent talks badly about people with or believed to have HIV; (6) if a respondent said people with or believed to have HIV lose respect from other people. If a respondent agreed or responded yes to the above six questions, it was coded as “0” and "1" otherwise. Then the total sum was recategorized as "No stigma" (score 6), "Low stigma" (scores 4 and 5), "Moderate stigma", (scores 2 and 3), and "High stigma" (score ≤ 1) [21].

#### Risky sexual behavior

Is generated by combining four questions about sexual behavior. Whether a woman had any STI in the last 12 months, a genital sore or ulcer in the last 12 months, genital discharge in the last 12 months, and at least one sexual partner other than her husband in the last 12 months The total sum was recategorized as "No risk" (score 0), "Some risk" (score 1), and "High risk" (score ≥ 2) [21].

#### Community-level media exposure

An aggregated variable from household media exposure measured as the proportion of women who had been exposed to at least one media (newspaper or magazine, radio, or television) and categorized based on the median value as low (communities with <50% of women exposed) and high (communities with ≥ 50% of women exposed).

#### Community-level maternal education

Aggregate values measured by the proportion of women with a minimum of primary education derived from data on respondents’ level of education and categorized using the median value of the proportion as low (communities with <50% of women have at least primary education) and high (communities with ≥ 50% of women have at least primary education).

### Data management and analysis

After accessing the data from the MEASURE DHS website, statistical software such as Excel, Stata version 16, and Arc-GIS version 10.8 were used for data extraction, re-coding, visualization, and other statistical analysis. Descriptive statistics were employed and presented as frequency, percentage, text, figures, and tables. Since the outcome variable was binary, logistic regression was expected to be applied. However, OR is a good approximation of PR when the outcome is rare [42]. Which means that when the prevalence is greater than 10%, the PR can be overestimated or underestimated by the OR [39, 40, 43].

Data from DHS is often hierarchical since observations from the same clusters are not independent, so the homoscedasticity assumption can be violated. A multilevel analysis is expected to explain clustering effects, which is why we used a multilevel generalized linear mixed effect model (Poisson regression with robust error variance) to identify associated factors. The median odds ratio (MOR) and Intra-class Correlation Coefficient (ICC) were used to measure an unexplained heterogeneity of the outcome across enumeration areas. Sampling weight (v005/1000000) was an adjustment factor applied to each case in tabulations to adjust for differences in the probability of selection and interview between cases in a sample due to either design or happenstance.

#### Descriptive spatial analysis

A spatial distribution analysis was conducted to highlight the characteristics of the spatial distribution of premarital HIV testing among currently married women. Then, a spatial autocorrelation analysis was conducted. Detecting spatial clustering in datasets plays an important role in spatial data analysis [44]. Different Global indices of spatial autocorrelation have been used to identify patterns of significant clustering. Global Moran’s I is a widely used global index that measures the similarity of values in neighboring places from an overall mean value and reflects a spatially weighted form of Pearson’s correlation coefficient [45]. The value ranges between -1 and 1. Values near − 1 indicate that the event was dispersed, whereas values near +1 indicate that the event was clustered and distributed randomly if Moran’s I value is zero. A statistically significant Moran’s I (p < 0.05) confirms the existence of spatial autocorrelation [46]. In order to determine if the pattern revealed by our data is clustered, dispersed, or random, we examined the spatial pattern of premarital HIV testing among married women in Ethiopia.

A hot spot analysis was conducted. Moran’s I statistics provide a summary of the spatial autocorrelation on a global scale, but local indicators of spatial autocorrelation like the Gettis-Ord Gi*statistic can give us more details by estimating the distribution of events at the local level and allowing us to study spatial relations in the study area of a specific observation [47]. The Gettis-Ord Gi* statistic determines statistically significant hot spots and cold spots by calculating a Z score and P value for each grid cell. Statistical significance is typically set at 99.9%, and it is typically expressed as the ratio of the total values in a certain location to the total values. The hot spot analysis was finally smoothed with the interpolation mapping technique using empirical Bayesian kriging kernel density estimate. As a result, the Bayesian technique is more frequently used to identify hot spots, since it can cut the number of false positives and false negatives by 50% when compared with conventional methods [48].

#### Spatial regression analysis

Spatial regression models are very crucial to understand the relationship between density of a certain events and other different environmental, demographic and socio-economic characteristics in the population [49]. Thus, we aimed to understand the relationship between prevalence of premarital HIV testing calculated at each cluster and other nine (9) explanatory variable from the multilevel analysis selected based on expert opinion and their significance during multilevel analysis.

We started our spatial regression modeling by OLS regression, which have assumptions like residuals needs to be independently and identically normally distributed. Besides, the residuals are assumed to be homoscedastic. There are many model diagnostics in OLS regression model such as R^2^, adjusted R^2^, VIF, Jarque-Bera statistic, Joint F statistic, Joint Wald statistic and Koenker (BP) statistic.

The R^2^ measure the amount of variation in outcome of interest explained by explanatory variables included in the model. Its value ranges between 0 and 1 where a model with R^2^ values closer to 1 has a better predictive performance. Adjusted R^2^ square is a similar measure however unlike R^2^ adjusted R^2^ cannot be influenced by the number of the variables included in the model which makes it a preferable measure [50]. The joint f and Wald statistics indicates the overall model significance and Wald statistics is preferable measure if Koenker statistic is significant (p<0.01). The Jarque Bera statistic indicates whether the model predictions are reliable. Significant Jarque Bera statistic indicates nonrandom model residuals. Koenker statistic is a measure of the consistency of the spatial process considered in the model. If it is significant it indicates that the spatial relationship is not consistent across areas due hetroscedasticity or non-stationarity. The variance inflation factors the presence of redundant variables in the model. All the diagnostics were checked.

Our OLS output advised us to ensure whether there is spatial autocorrelation in the residuals and we carried out the spatial autocorrelation test to determine if the residuals are auto-correlated. The result showed that residuals were sufficiently autocorrelated thus the OLS regression analysis was unreliable. The modeling process is spatially heterogeneous or non-stationary. Thus, it is necessary to make reliable predictions by using GWR.

The GWR was conducted with similar dependent and explanatory variables considered in the global model. The GWR have a geographical weighting system to the features included in the local regression equations. Near features and features that are farther away from the regression point have more weight and less weight in the regression equation respectively. These weights are determined by a distance decay function called kernel [51]. In ArcGIS there are two kernel types, fixed and adaptive. The spatial configuration of the feature is a main reason to choose the kernel type. For reasonably or regularly positioned observations fixed kernel is appropriate. However, if observations are clustered an adaptive kernel is appropriate [50].

Another most important parameter to be considered in GWR is a bandwidth (neighborhood). It is the distance band or number of neighbors used for each local regression equation and which controls the degree of smoothing in the model [51]. In ArcGIS There are three choices for the corrected Akaike Information Criterion (AICc), Cross Validation (CV) and Bandwidth Parameter. The AICc method automatically finds a bandwidth with minimum the AICc, while the CV finds a bandwidth which minimizes a Cross Validation score. In practice there isn’t much to choose between the two methods, although the AICc is our preferred method. The AICc method can reduce model complexity due to number of variables included in the study and the bandwidth [50].

In our study, observation showed a significant clustering and the explanatory variables include in the model were nine. Thus, in order to reduce model complexity, we were interested to use adaptive kernel type determined by AICc.

Model comparison between OLS and the GWR model was made using adjusted R^2^ and AICc value. A model with high value of adjusted R^2^ and low AICc value was the preferred model (GWR). If we get less than 4 AICc difference between two models we cannot choose one of them, but if this difference is greater than 10 there is evidence to choose a model with small AICc value [52]. Finally the spatial autocorrelation test was conducted among residuals of the GWR model to determine whether the residuals are randomly distributed and after we controlled the spatial dependencies present in the residuals for the OLS.

### Model building process

The model building process began with an empty Generalized Linear Mixed Model and complex models were built step by step. Four model displayed in our analysis; model-I (null model), model II (containing only individual factors), model III (containing only community factors) and model IV (containing both individual and community-level factors). Model fitness was assessed based on log likelihood Ratio (LL) and information criteria’s (AIC and BIC). Bi-variable and multivariable two level robust Poisson regression model were fitted to identify determinant factors. Finally adjusted prevalence ratio (APR) with a 95% Confidence Interval (CI) and p-value ≤ 0.05 in the selected multivariable model was used to declare significant factors.

### Missing values

Any missing data in the dataset was managed according to the DHS guidelines. Thus, the final model was based on complete observation.

### Ethical considerations

The study was a secondary data analysis based on the publicly available DHS datasets. According to the 2016 report, data was gathered after participants gave their formal consent to be tested for HIV. The protocol for drawing blood samples and analyzing them was based on the anonymous linked protocol created for the DHS Program, which permits the combination of HIV test results with the sociodemographic information gathered from individual questionnaires after all information that could be used to identify a specific person has been removed. On February 1, 2023, the MEASURE DHS program sent us an official authorization letter to download and use the data for our study, which we have attached.

## Results

### Characteristics of the respondents

Our study included a total of 10,223 weighted samples of married reproductive-age women. Of those, about 23.5% were in the age group of 25–29. About 61.2% reported that they had not attended formal education. More than four-fifths (83.8%) were living in rural areas of Ethiopia, while more than one-third of the participants were living in the Oromia region. About 61.7% and 20.9% were from households without media exposure and the richest wealth quintile, respectively. More than three-fourths (76.4%) reported that they knew the place where HIV testing is provided. Most (85.2%) had a history of khat chewing, while about 65.3% had not drunk any alcohol-containing drinks. More than half (51.8%) had a previous history of HIV testing. About 38.9% had a moderate level of HIV-related perceived stigma, while almost four-fifths (79.7%) had low HIV-related knowledge. Most (95.5%) had no history of risky sexual behavior. More than half (54.6%) of the participants reported that the distance between their house and the health facility was a big problem (**Table 1**).

**Table 1:** Characteristics of the respondents, EDHS 2016.

Variables	Categories	Weighted frequency(10,223)	Percentage (%)
Maternal age	15–19	588	5.8
	20–24	1, 710	16.7
	25–29	2, 402	23.5
	30–34	2, 049	20.0
	35–39	1, 613	15.8
	40–44	1, 064	10.4
	45–49	798	7.8
Education level	No education	6, 253	61.2
	Primary	2, 895	28.3
	Secondary	654	6.4
	Higher	421	4.1
Religion	Orthodox	4, 139	40.5
	Catholic	75	0.7
	Protestant	2, 289	22.4
	Muslim	3, 540	34.6
	Other	179	1.8
Household media exposure	No	6, 309	61.7
	Yes	3, 914	38.3
Wealth index	Poorest	1, 953	19.1
	Poorer	2, 074	20.3
	Middle	2, 057	20.1
	Richer	1, 999	19.6
	Richest	2, 140	20.9
knowing the place of HIV testing	No	2, 413	23.6
	Yes	7, 810	76.4
Khat chewing	No	8, 710	85.2
	Yes	1, 514	14.8
Alcohol drinking	No	6, 679	65.3
	Yes	3, 545	34.7
Previous history of HIV testing	No	5, 300	51.8
	Yes	4, 924	48.2
HIV related stigma	No	1, 204	11.8
	Low	2, 874	28.1
	Moderate	3, 976	38.9
	High	2, 168	21.2
HIV related knowledge	Low	8, 150	79.7
	High	1, 887	18.5
	Comprehensive	186	1.8
Employment	Working	3, 163	30.9
	Not working	7, 060	69.1
Visited health facility	No	5, 224	51.1
	Yes	4, 999	48.9
Age at first sex	< 20	8, 503	83.2
	≥ 20	1,719	16.8
Recent sexual activity	Never	6	0.1
	Active last 4 weeks	8, 224	80.4
	Not active last 4 weeks	1, 993	19.5
History of risky sexual behaviors	No risk	9, 766	95.5
	Some risk	365	3.6
	High risk	93	0.9
Residence	Urban	1, 658	16.2
	Rural	8, 565	83.8
Region	Tigray	658	6.4
	Afar	96	0.9
	Amhara	2, 414	23.6
	Oromia	3, 987	39.0
	Somali	324	3.2
	Benishangul	114	1.1
	SNNPR	2, 173	21.3
	Gambela	29	0.3
	Harari	25	0.2
	Addis Ababa	355	3.5
	Dire Dawa	50	0.5
Distance to health facility	Big problem	5, 582	54.6
	Not big problem	4, 641	45.4
Community-level maternal education	Low	6, 100	59.7
	High	4,123	40.3
Community level media exposure	Low	5, 838	57.1
	High	4, 385	42.9

### Magnitude of premarital HIV testing

**Fig 1:**
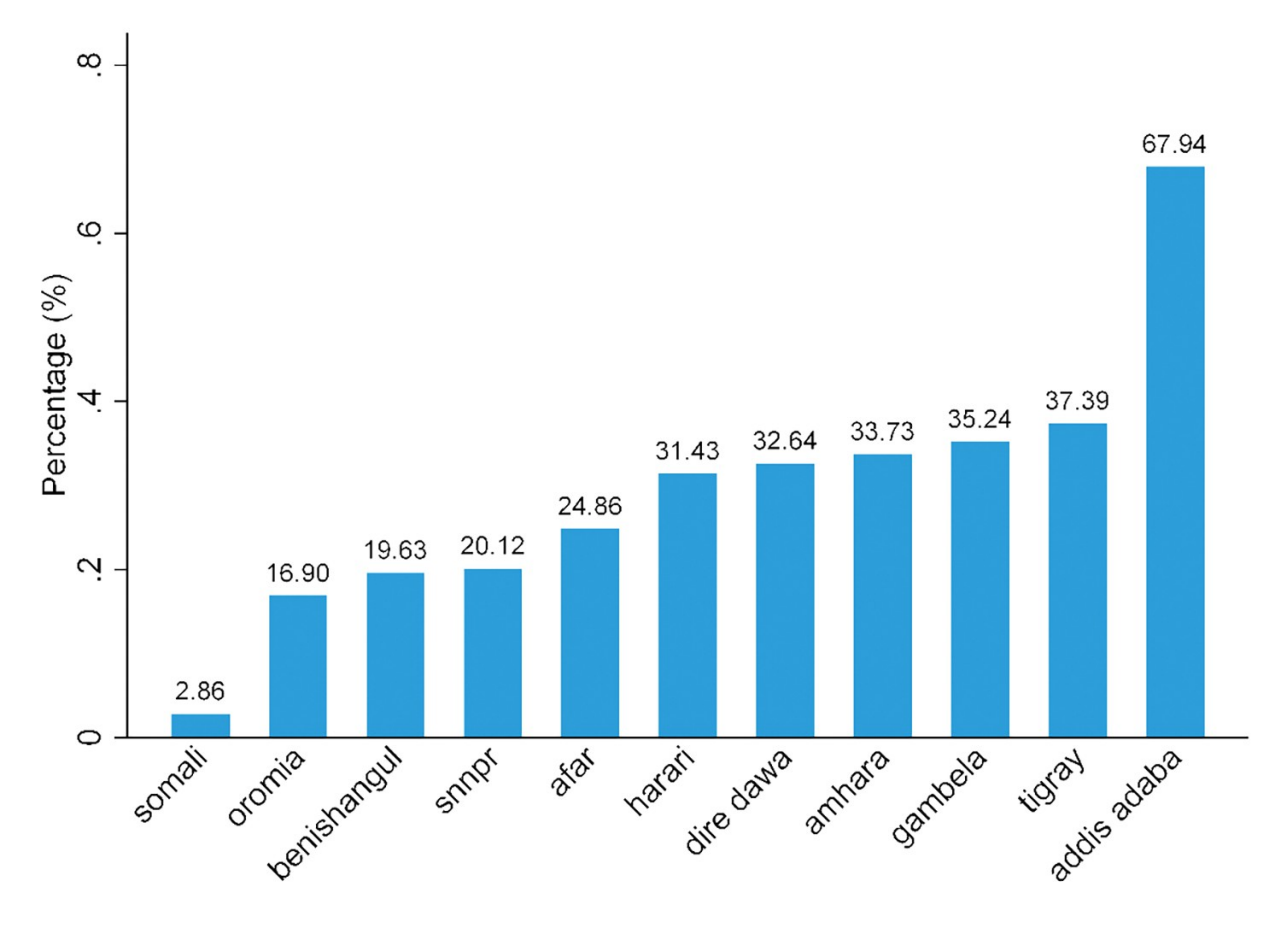
Prevalence of premarital HIV testing across regions of Ethiopia, 2016.

The prevalence of premarital HIV testing was disproportionately distributed across regions of Ethiopia, with the highest prevalence observed in Addis Ababa (67.94%), the lowest in Somali (2.86%), and an overall prevalence of 24.5% [95% CI: 23.65–25.32%] (**Fig 1**).

## Factors associated with premarital HIV testing

### Random effect analysis

The calculated ICC was determined to be 44.1% [95% CI: 40.03–48.24%]. Thus, 44.1% of the variation in premarital HIV testing has been attributed to the clustering effect alone. The MOR was 4.65 with a [4.05, 5.25] 95% Credible Interval. Moving a woman from a cluster with low premarital HIV testing prevalence to one with high prevalence can raise the probability of premarital HIV testing by 4.65 fold. As a result, we concluded that the multilevel mixed effect model was preferable to the conventional model. Subsequently, we examined the Null model, which has no predictor variables, Model II, which has factors at the individual level, Model III, which has factors at the community level, and Model IV, which has factors at both the individual and community levels. Finally, model IV was selected because it had a smaller AIC and deviance (**Table 2**).

**Table 2:** Model comparison among candidate multilevel robust poisson regression models.

Model	LL	Deviance (-2LL)	AIC
Null model (I)	-5607.34	11214.68	11218.67
Model–II	-4740.60	9481.20	9551.21
Model–III	-5268.30	10536.6	10568.60
Model–IV	-4679.40	9358.80	9456.81

### Fixed effect analysis (multilevel robust poisson regression)

Premarital HIV testing had a significant association with factors such as women’s age, educational level, religion, household media exposure, household wealth index, history of khat use, history of HIV testing, HIV-related stigma, HIV-related knowledge, age at first sex, distance to a health facility, region, and community-level media exposure.

Women in the age groups 20–24, 25–29, and 30–34 had a 16% [APR = 0.84, 95% CI: 0.77, 0.92], 28% [APR = 0.72, 95% CI: 0.65, 0.80], and 39% [APR = 0.61, 95% CI: 0.54, 0.68] lower prevalence of premarital HIV testing. Women in the age groups of 35–39, 40–44, and 45–49 had a 56% [APR = 0.44, 95% CI: 0.38, 0.50], 68% [APR = 0.32, 95% CI: 0.27, 0.38], and 66% [APR = 0.34, 95% CI: 0.27, 0.43] lower prevalence of premarital HIV testing compared to women in the 15–19 age group.

Formally educated women were more likely to have premarital HIV testing. Among women with primary, secondary, or higher levels of education, there was a 31% [APR = 1.31, 95% CI: 1.19, 1.43], a 49% [APR = 1.49, 95% CI: 1.35, 1.65], and a 47% [APR = 1.47, 95% CI: 1.33, 1.64] higher prevalence of premarital HIV testing compared with those without formal education, respectively. In comparison to women who follow orthodox religion, prevalence of premarital HIV testing was decreased in Protestant and other religion-following women by 15% [APR = 0.85, 95% CI: 0.76, 0.96] and 51% [APR = 0.49, 95% CI: 0.26, 0.91], respectively. The prevalence of premarital HIV testing was 23% higher among women from media-exposed homes compared to their counterparts [APR = 1.23; 95% CI: 1.12, 1.36]. In comparison to women from households in the lowest wealth quintile, there was a 29% [APR = 1.29, 95% CI: 1.09, 1.53] increase in the prevalence of premarital HIV testing among women from households in the highest wealth quintile.

In comparison to their counterparts, women who had ever consumed khat had a 16% higher prevalence of premarital HIV testing [APR = 1.16; 95% CI: 1.06, 1.28]. Compared to their counterparts, women with a prior experience of HIV testing had a 4.09 times higher prevalence of premarital HIV testing (APR = 4.09, 95% CI: 3.44, 4.88). Women with a high perceived HIV-related stigma had a 21% decreased prevalence of premarital HIV testing [APR = 0.79, 95% CI: 0.68, 0.90] compared to those without perceived HIV-related stigma. Comparing women with low levels of HIV-related knowledge, those with comprehensive HIV-related knowledge exhibited a 48% [APR = 1.48; 95% CI: 1.09, 2.01] increased prevalence in premarital HIV testing.

The prevalence of premarital HIV testing was 9% higher [APR = 1.09, 95% CI: 1.09, 2.01] among women who reported no significant difficulty traveling to a health institution compared to those who reported the issue. Women who had experienced their first sexual intercourse before the age of 20 years had a 20% [APR = 0.80, 95% CI: 0.76, 0.85] lower prevalence of premarital HIV testing compared to their counterparts. Women who were residing in the Amhara region had a 17% [APR = 1.17, 95% CI: 1.00, 1.36] higher prevalence of premarital HIV testing compared to women in the Tigray region. While women who were residing in Oromia, Somali, Benishangul, Harari, and Dire Dawa regions had a 20% [APR = 0.80, 95% CI: 0.67, 0.96], 73% [APR = 0.27, 95% CI: 0.17, 0.45], 24% [APR = 0.76, 95% CI: 0.60, 0.97], 28% [APR = 0.72, 95% CI: 0.60, 0.85], and 26% [APR = 0.74, 95% CI: 0.61, 0.89] lower prevalence of premarital HIV, testing respectively. Having high community-level media exposure showed a 14% [APR = 1.14, 95% CI: 1.01, 1.30] increased prevalence of premarital HIV testing (**Table 3**).

**Table 3:** Bi-variable and multivariable multilevel mixed-effect robust poisson regression analysis of premarital HIV testing among married women in Ethiopia, using 2016 EDHS.

Variables	Categories	Crude PR[95% CI]	Adjusted PR[95% CI]
Maternal age	15–19	1.00	1.00
	20–24	0.98 [0.88, 1.09]	0.84 [0.77, 0.92] **
	25–29	0.80 [0.71, 0.90] **	0.72 [0.65, 0.80] **
	30–34	0.61 [0.54, 0.70] **	0.61 [0.54, 0.68] **
	35–39	0.41 [0.35, 0.47] **	0.44 [0.38, 0.50] **
	40–44	0.29 [0.24, 0.36] **	0.32 [0.27, 0.38] **
	45–49	0.27 [0.21, 0.35] **	0.34 [0.27, 0.43] **
Education level	No education	1.00	1.00
	Primary	2.60 [2.34, 2.89] **	1.31 [1.19, 1.43] **
	Secondary	4.06 [3.59, 4.59] **	1.49 [1.35, 1.65] **
	Higher	4.58 [4.03, 5.22] **	1.47 [1.33, 1.64] **
Religion	Orthodox	1.00	1.00
	Catholic	0.85 [0.55, 1.32]	0.88 [0.60, 1.30]
	Protestant	0.74 [0.65, 0.84] **	0.85 [0.76, 0.96] *
	Muslim	0.62 [0.54, 0.70] **	1.00 [0.91, 1.11]
	Other	0.25 [0.11, 0.55] **	0.49 [0.26, 0.91]*
Media exposure	No	1.00	1.00
	Yes	2.50 [2.22, 2.81] **	1.23[1.12, 1.36] **
Wealth index	Poorest	1.00	1.00
	Poorer	1.50 [1.24, 1.81] **	1.01 [0.87, 1.19]
	Middle	1.83 [1.51, 2.22] **	1.12 [0.98, 1.30]
	Richer	2.36 [1.95, 2.85] **	1.14 [0.99,1.32]
	Richest	4.90 [4.07, 5.89] **	1.29 [1.09, 1.53]*
knowing the place of HIV testing	No	1.00	1.00
	Yes	4.79 [3.79, 6.05] **	1.31 [1.00, 1.71]
Khat chewing	No	1.00	1.00
	Yes	1.08 [0.98, 1.19]	1.16 [1.06, 1.28] *
Alcohol drinking	No	1.00	1.00
	Yes	1.25 [1.16, 1.36] **	1.02 [0.95, 1.10]
Previous history of HIV testing	No	1.00	1.00
	Yes	7.99 [6.78, 9.41] **	4.09 [3.44, 4.88] **
HIV related stigma	No	1.00	1.00
	Low	1.31 [1.16, 1.46] **	0.94 [0.86, 1.04]
	Moderate	1.24 [1.10, 1.39] **	0.94 [0.85, 1.03]
	High	0.84 [0.72, 0.99]*	0.79 [0.68, 0.90]**
HIV related knowledge	Low	1.00	1.00
	High	1.04 [0.96, 1.13]	1.06 [0.98, 1.14]
	Comprehensive	0.96 [0.65, 1.41]	1.48 [1.09, 2.01]*
Employment	Working	1.19 [1.12, 1.28] **	1.04 [0.98, 1.09]
	Not working	1.00	1.00
Visited health facility	No	1.00	1.00
	Yes	1.42 [1.32, 1.52] **	1.01 [0.95, 1.08]
Age at first sex	< 20	0.68 [0.63, 0.72] **	0.80 [0.76, 0.85]**
	≥ 20	1.00	1.00
Recent sexual activity	Never	1.53 [0.78, 3.00]	1.06 [0.61, 1.86]
	Active last 4 weeks	1.06 [0.98, 1.14]	1.04 [0.98, 1.11]
	Not active last 4 weeks	1.00	1.00
Risky sexual behaviors	No risk	1.00	1.00
	Some risk	1.12 [0.98, 1.28]	0.96 [0.85, 1.09]
	High risk	0.98 [0.74, 1.29]	0.89 [0.68, 1.17]
Community level variables			
Residence	Urban	1.00	1.00
	Rural	0.28 [0.24, 0.31] **	0.96[0.86, 1.07]
Region	Tigray	1.00	1.00
	Afar	0.46 [0.32, 0.67] **	1.01 [0.80, 1.28]
	Amhara	0.87 [0.68, 1.11]	1.17 [1.00, 1.36] *
	Oromia	0.45 [0.34, 0.59] **	0.80 [0.67, 0.96] *
	Somali	0.08 [0.04, 0.14] **	0.27 [0.17, 0.45] **
	Benishangul	0.44 [0.30, 0.62] **	0.76 [0.60, 0.97]*
	SNNPR	0.55 [0.42, 0.72] **	0.95 [0.78, 1.16]
	Gambela	0.73 [0.55, 0.97]*	0.93 [0.80, 1.09]
	Harari	0.89 [0.64, 1.24]	0.72 [0.60, 0.85] **
	Addis Ababa	1.93 [1.59, 2.35] **	0.97 [0.85, 1.10]
	Dire Dawa	0.89 [0.63, 1.24]	0.74 [0.61, 0.89] **
Distance to health facility	Big problem	1.00	1.00
	Not big problem	1.55 [1.41, 1.71] **	1.09 [1.01, 1.17]*
Community-level maternal education	Low	1.00	1.00
	High	3.63 [3.11, 4.23] **	1.07 [0.95, 1.21]
Community-level media exposure	Low	1.00	1.00
	High	3.77 [3.25, 4.38] **	1.14 [1.01, 1.30]*

#### Spatial distribution premarital HIV testing

**Fig 2:**
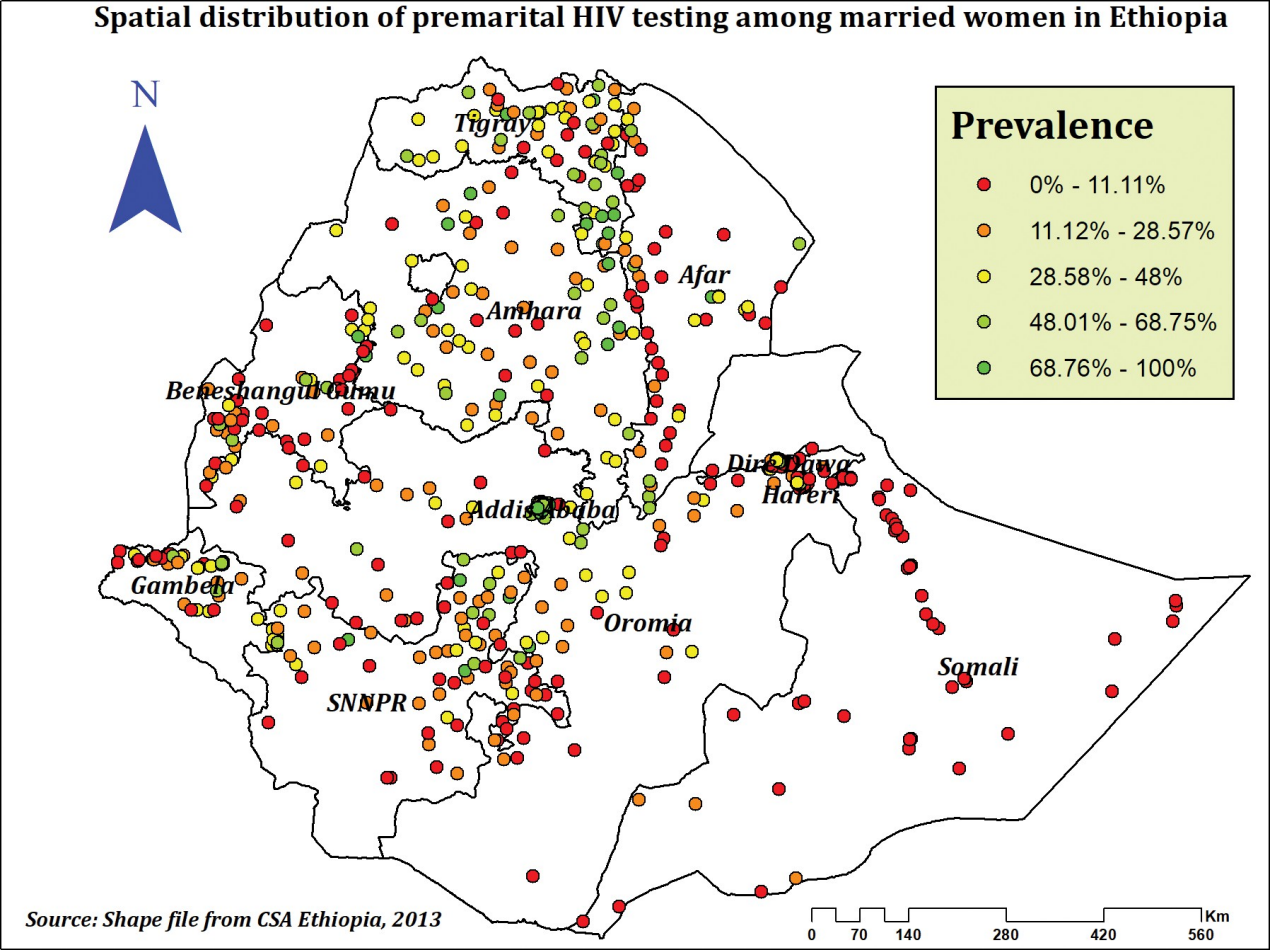
Spatial distribution of premarital HIV testing among married women in Ethiopia, using 2016 EDHS.

**Fig 3:**
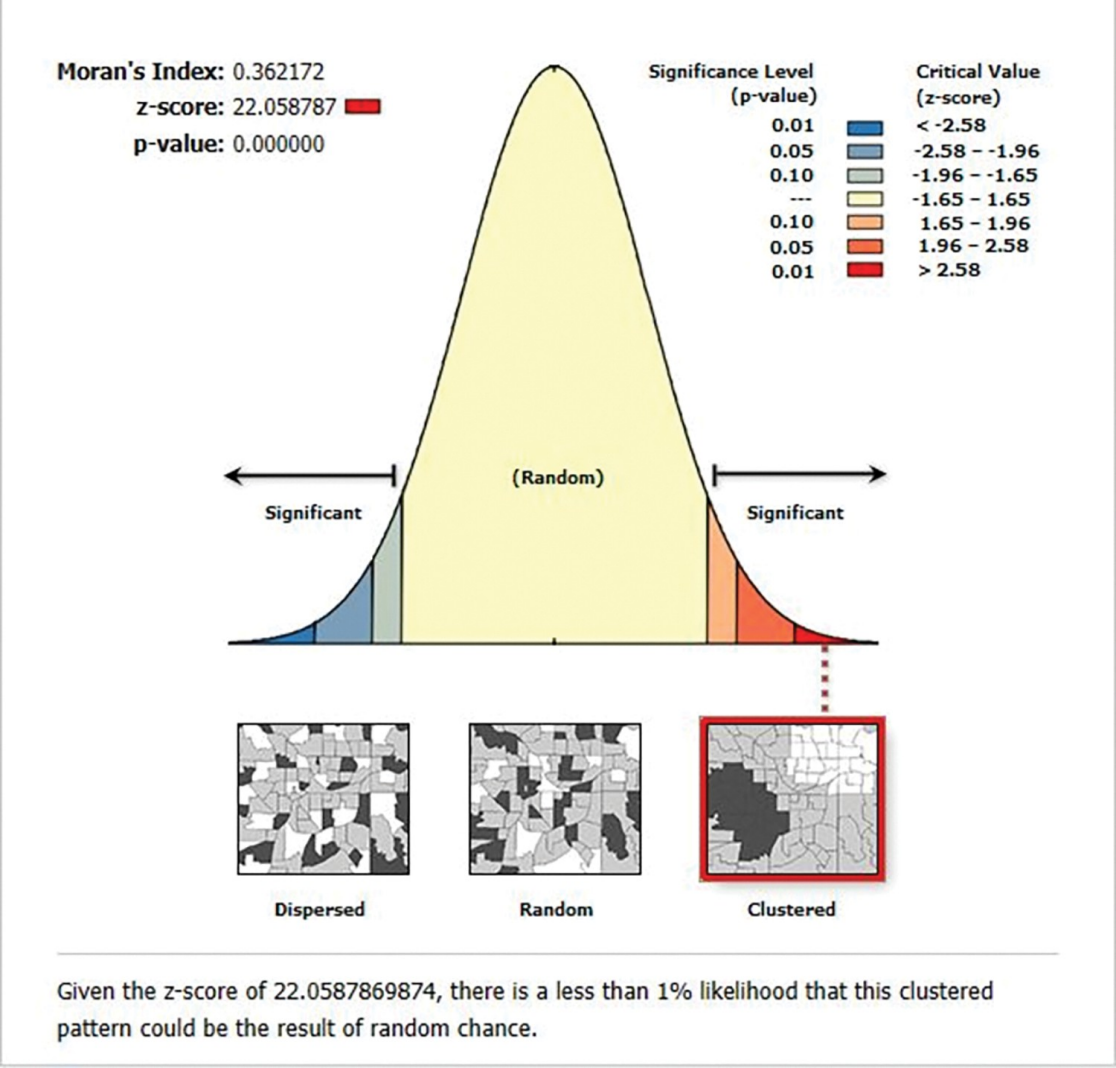
Result of the global spatial autocorrelation analysis of premarital HIV testing among married women in Ethiopia, using 2016 EDHS.

**Fig 4:**
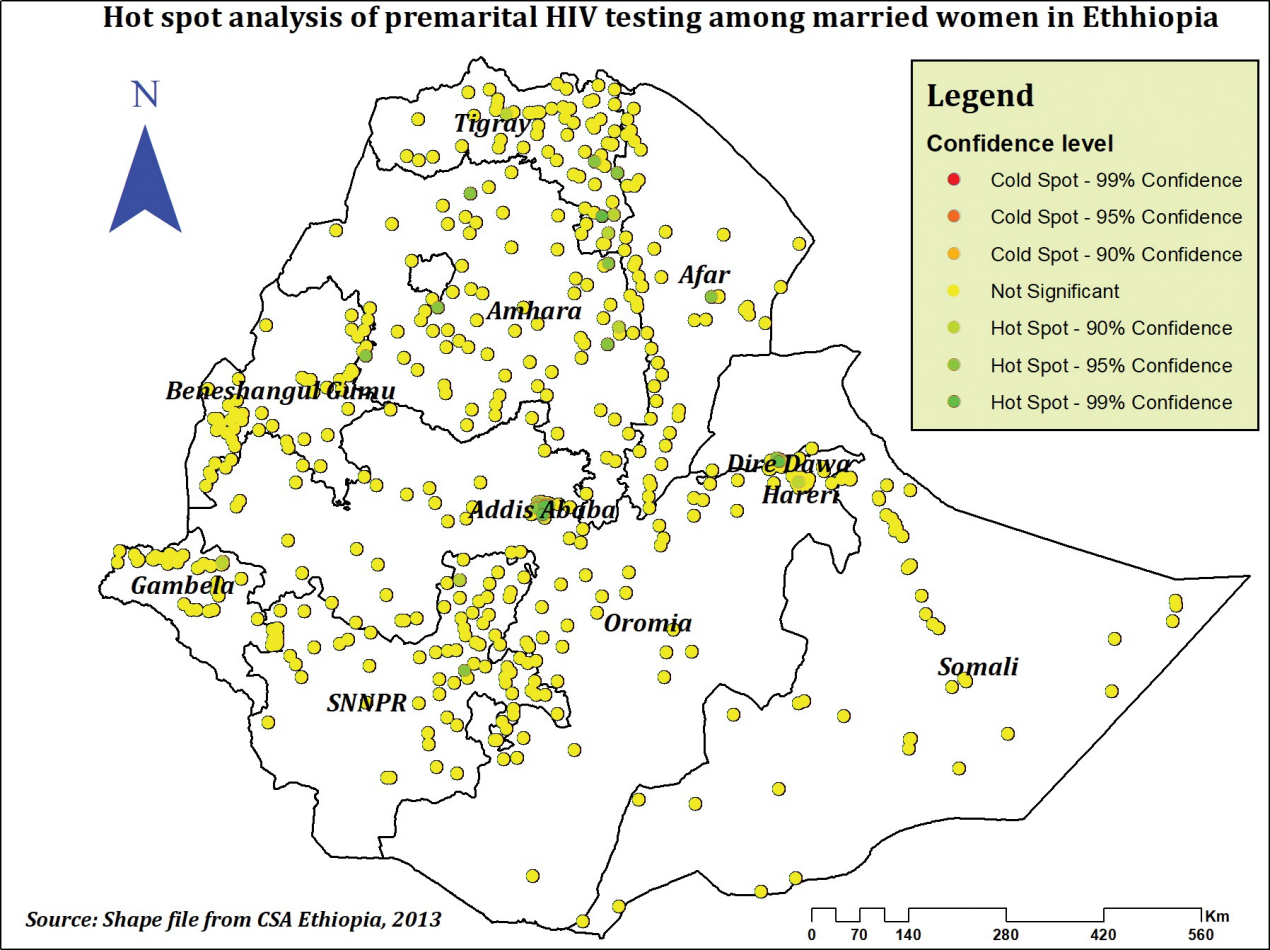
Hot spot analysis of premarital HIV testing among married women in Ethiopia, using 2016 EDHS.

The highest prevalence of premarital HIV testing was observed in Addis Ababa, Tigray, and Amhara regions, whereas the lowest prevalence was observed in the majority of Somali, Afar, Oromia, and Gambela regions (**Fig 2**). Premarital HIV testing was significantly spatially clustered across the country, according to the spatial autocorrelation analysis, which found a significant global Moran’s I of 0.36 (P-value = 0.000, Z-score of 22.06)(**Fig 3**). This suggests that the observed Moran’s I is higher than the expected Moran’s I and establishes that the feature has nearby features with similar high qualities or values. The Getis-Ord Gi* revealed substantial hot spot areas during hot spot analysis in Addis Ababa, Dire Dawa, North Tigray, and some sections of the Afar and Amhara regions (**Fig 4**).

### Spatial prediction of premarital HIV testing

**Fig 5:**
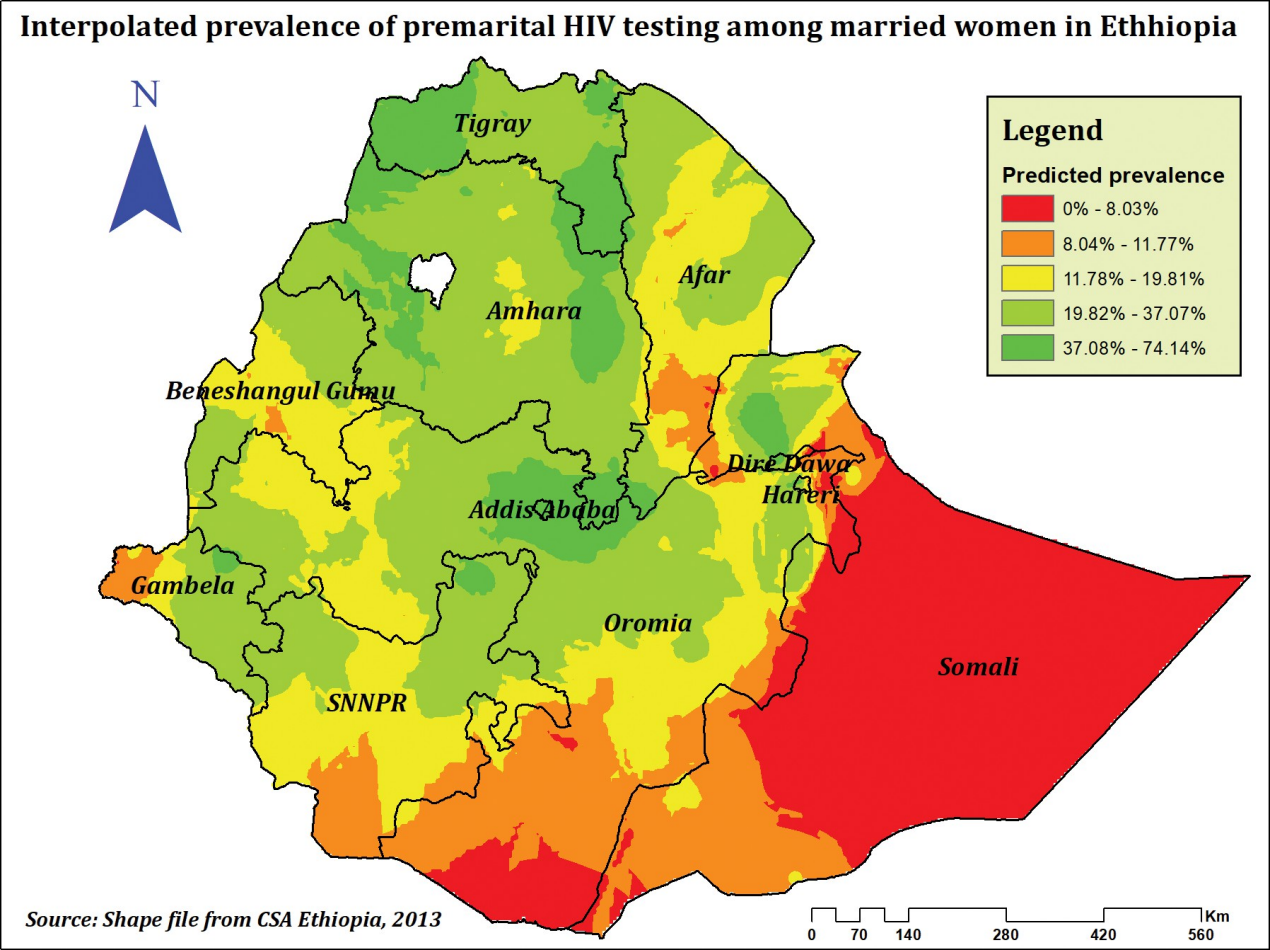
Interpolated prevalence of premarital HIV testing among currently married women in Ethiopia, using 2016 EDHS.

The locations of high and low prevalence for premarital HIV testing among married women in Ethiopia were predicted using empirical Bayesian kriging interpolation techniques. The majority of Somalia, the southern parts of Oromia, western Gambela, some parts of Afar, and the Benishangul Gumuz region were predicted to have a low prevalence of premarital HIV testing, whereas the entire Tigray, Amhara, Addis Ababa, some parts of Oromia, some parts of SNNP, and some parts of Gambela and Afar were predicted to have a high prevalence **(Fig 5).**

#### The ordinary least square regression analysis results

The joint F and Wald statistics of the OLS model were significant, indicating that the model is significant overall (it has at least one significant variable that accounts for the variation in premarital HIV testing). The Jarque Bera statistic was significant (P = 0.000), indicating that the model’s predicted values had been biased and that the residuals weren’t distributed randomly over the areas. The Breusch-Pagan hetroscedasticity coefficient given by the Koenker statistic was significant (p = 0.005)(**Table 4)**. It suggests that due to non-stationarity or hetroscedasticity, there is an inconsistent relationship between the prevalence of premarital HIV testing and spatial explanatory variables.

**Table 4:** OLS regression analysis results, EDHS 2016.

Variable		Coefficient	Robust Std.error	Robust probability	VIF
Intercept		-0.08	0.038	0.045	----
Proportion of women with secondary and above education		0.26	0.043	**0.000**	2.39
Proportion of women from household with richer and above wealth quintile		0.08	0.033	**0.016**	3.92
Proportion of women from household with media exposure		0.23	0.038	**0.000**	3.95
Proportion of women with a big problem of distance to health facility		-0.09	0.026	**0.001**	1.89
Proportion of women with high and comprehensive HIV related knowledge.		0.10	0.054	0.076	1.25
Proportion of women with moderate and high HIV related stigma.		-0.01	0.029	0.713	1.32
Proportion of women with high risky sexual behaviour		-0.54	0.164	**0.001**	1.01
Proportion of women who had ever chewed khat		0.02	0.035	0.511	1.09
Proportion of women who knows place of HIV testing		0.27	0.038	**0.000**	1.67
**OLS diagnostics**					
**Diagnostic parameters**	**Value**			**P-value**	
Number of observations	622			---	
Joint F statistic	126.33			**0.000**	
Joint Wald statistic	1288.71			**0.000**	
Koenker(BP) statistic	23.50			**0.005**	
Jarque Bera statistic	56.96			**0.000**	
**Model comparison**					
**Model parameter**	**OLS (global) model**			**GWR**	
R^2^	65.01			71.66	
Adjusted R^2^	64.49			69.19	
AICc	-520.79			-588.39	

^a^Std.error- Standard Error, VIF-Variance Inflation Factor, OLS-Ordinary Least Square, GWR-Geographically Weighted Regression.

One of the assumptions of OLS regression analysis was violated since the spatial autocorrelation of the OLS model’s residuals was not independent and identically distributed (significantly autocorrelated). The OLS regression’s findings are therefore untrustworthy. We require GWR to account for the spatial autocorrelation and varying relationships across space, increase the accuracy of the predictions, and map area-specific coefficient estimates of explanatory variables that explain the heterogeneity. The Variance Inflation Factor (VIF) evaluates whether explanatory variables taken into account in the model are redundant or collinear. According to a general guideline, the cutoff value for VIF is 7.5, hence any explanatory variable with a VIF value higher than 7.5 needs to be eliminated [53]. Multicolinearity was not a significant issue in the subsequent analysis because all explanatory variables in our OLS model had a VIF value lower than 7.5.

In the global model, hot spot areas of premarital HIV testing among married women were significantly associated with secondary and above education levels, richer and above household wealth quintiles, households media exposure, a significant problem of distance to health facilities, high-risk sexual behavior, and knowledge of place of HIV testing (**Table 4**).

#### Geographical weighted regression

**Fig 6:**
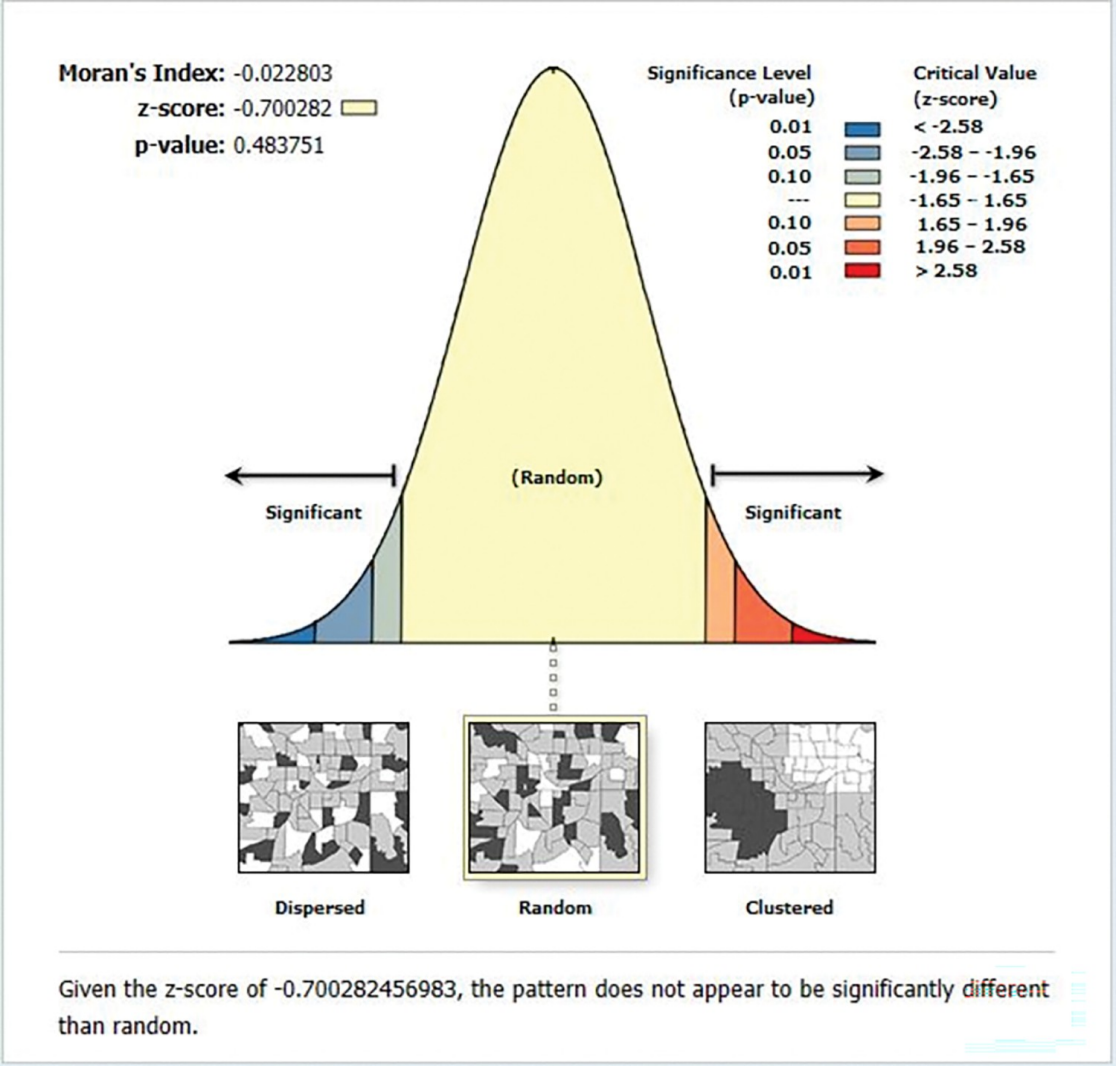
The global spatial autocorrelation analysis of residuals of the geographical weighted regression model.

Similar explanatory factors indicated for the OLS model were used for the GWR analysis. The GWR model showed an improvement over the OLS model. The adjusted R^2^ was raised from 64.49 in the OLS to 69.19 in the GWR model. Which implies that an additional 4.7% of the variation in premarital HIV testing prevalence across regions was explained by the GWR model. Additionally, the GWR’s AICc value was -580.39 whereas the OLS model’s AICc value was -520.79 (difference: 59.6)(**Table 4**). So, it is clear that the GWR model adequately accounted for the spatial heterogeneity. We examined the spatial autocorrelation of the residuals before interpreting the results from the GWR. Since the residuals’ autocorrelation was distributed randomly after GWR, Moran’s I for the residuals was -0.70 (p = 0.48). In **Fig 6**, there is little indication of autocorrelation, indicating that the explanatory variables included in our model successfully described the spatial dependencies found in the residuals from the OLS model and that our model was well specified. Regarding the direction of influence on our outcome of interest, the coefficients from the global model and the coefficients from the GWR model were in agreement.

**Fig 7:**
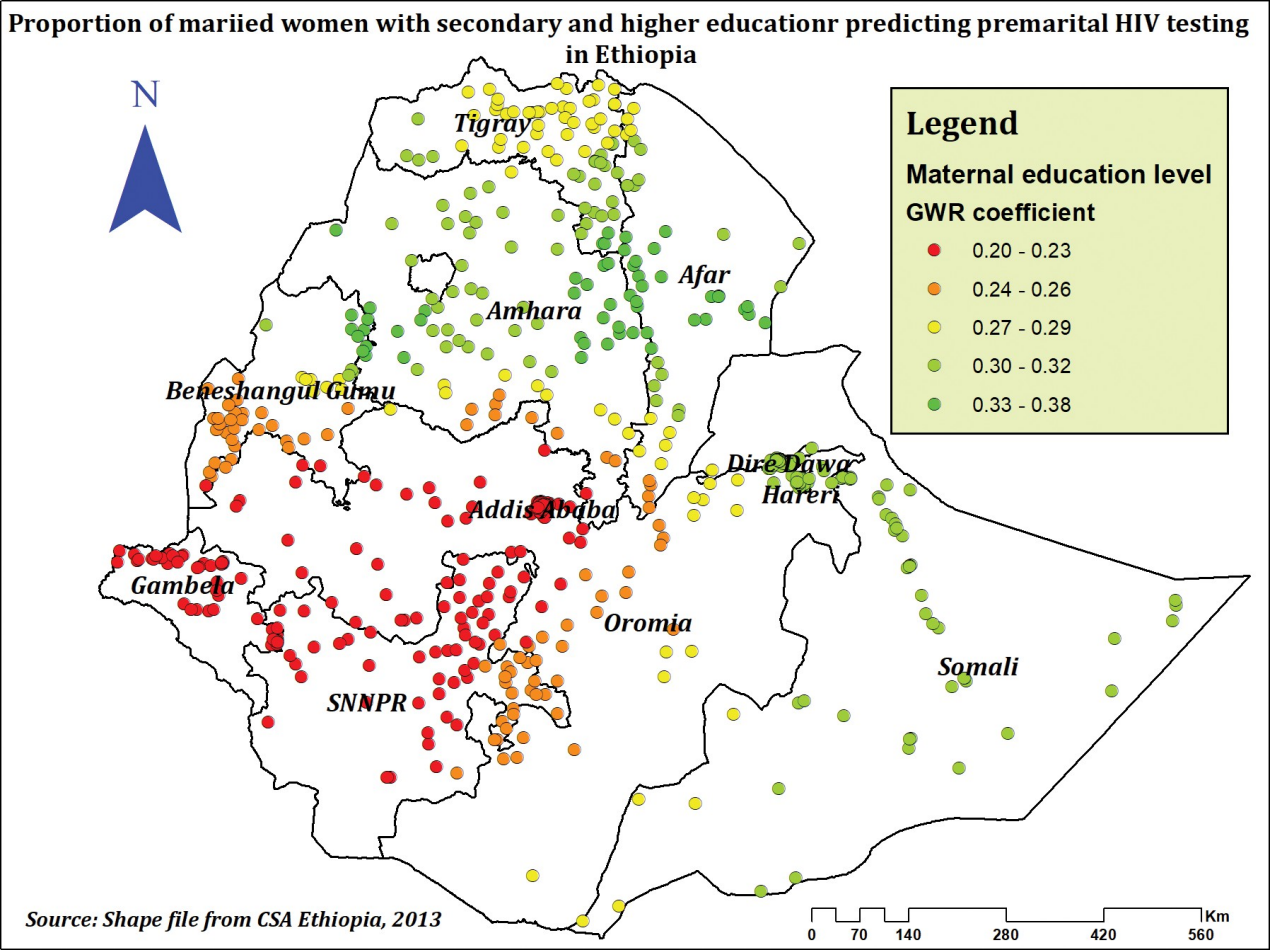
The GWR coefficients of secondary and above women education level predicting premarital HIV testing among married women in Ethiopia, using 2016 EDHS.

The proportion of women with secondary and higher education levels and premarital HIV testing hot spot areas were found to have positive associations in our final model (GWR). The prevalence of premarital HIV testing among married women significantly rises in the Amhara, Afar, and Somali regions as the percentage of women with secondary and higher education rises. (**Fig 7**).

**Fig 8:**
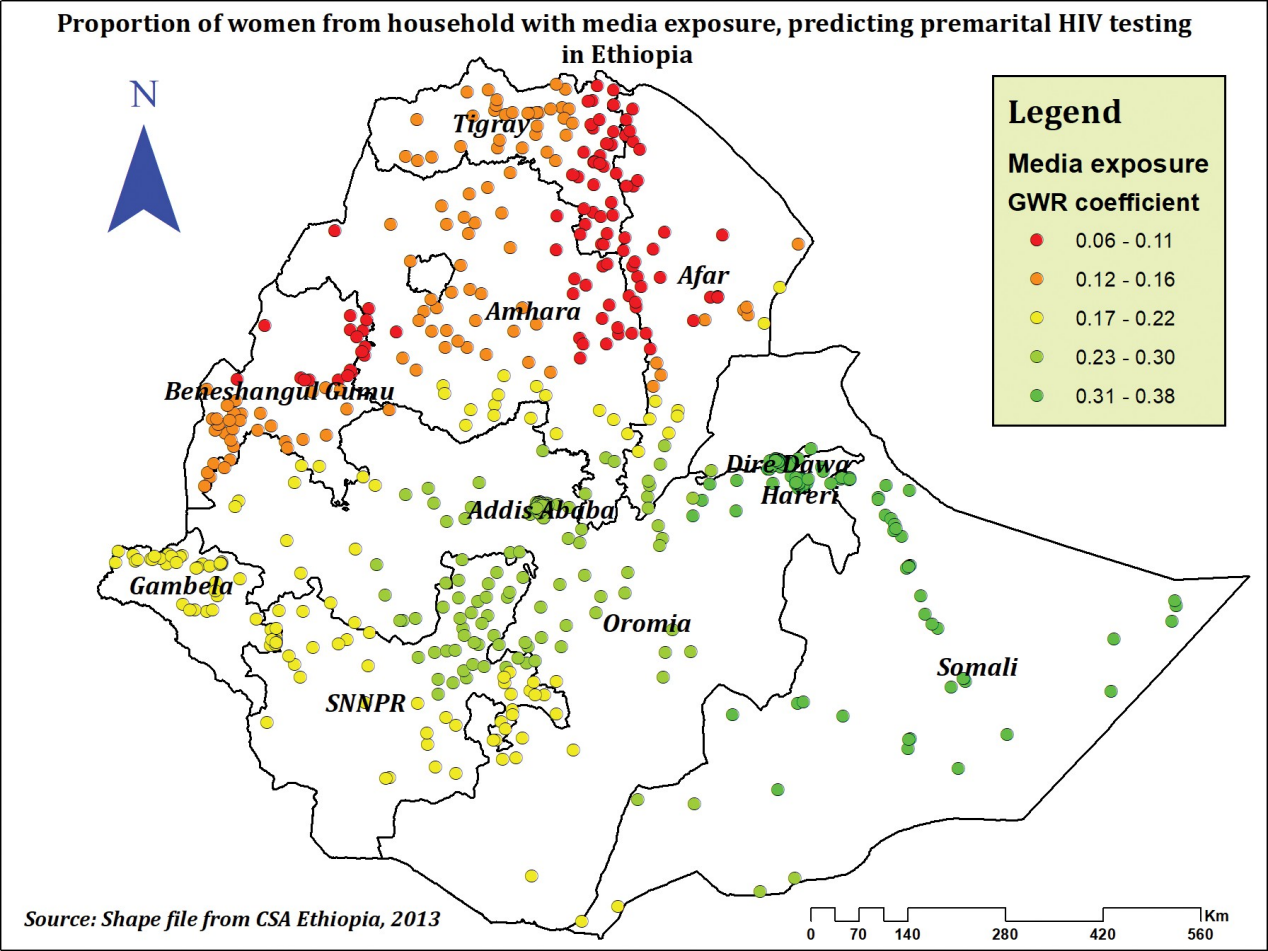
The GWR coefficients of household media exposure predicting premarital HIV testing among married women in Ethiopia, using 2016 EDHS.

Premarital HIV testing prevalence and the percentage of women who have access to media in their homes were positively related. The prevalence of premarital HIV testing was highly increased in Addis Ababa, Dire Dawa, Hareri, Somali, some parts of Oromia, some parts of the SNNPR, and some parts of the Afar regions as the percentage of women exposed to media increased (**Fig 8**).

**Fig 9:**
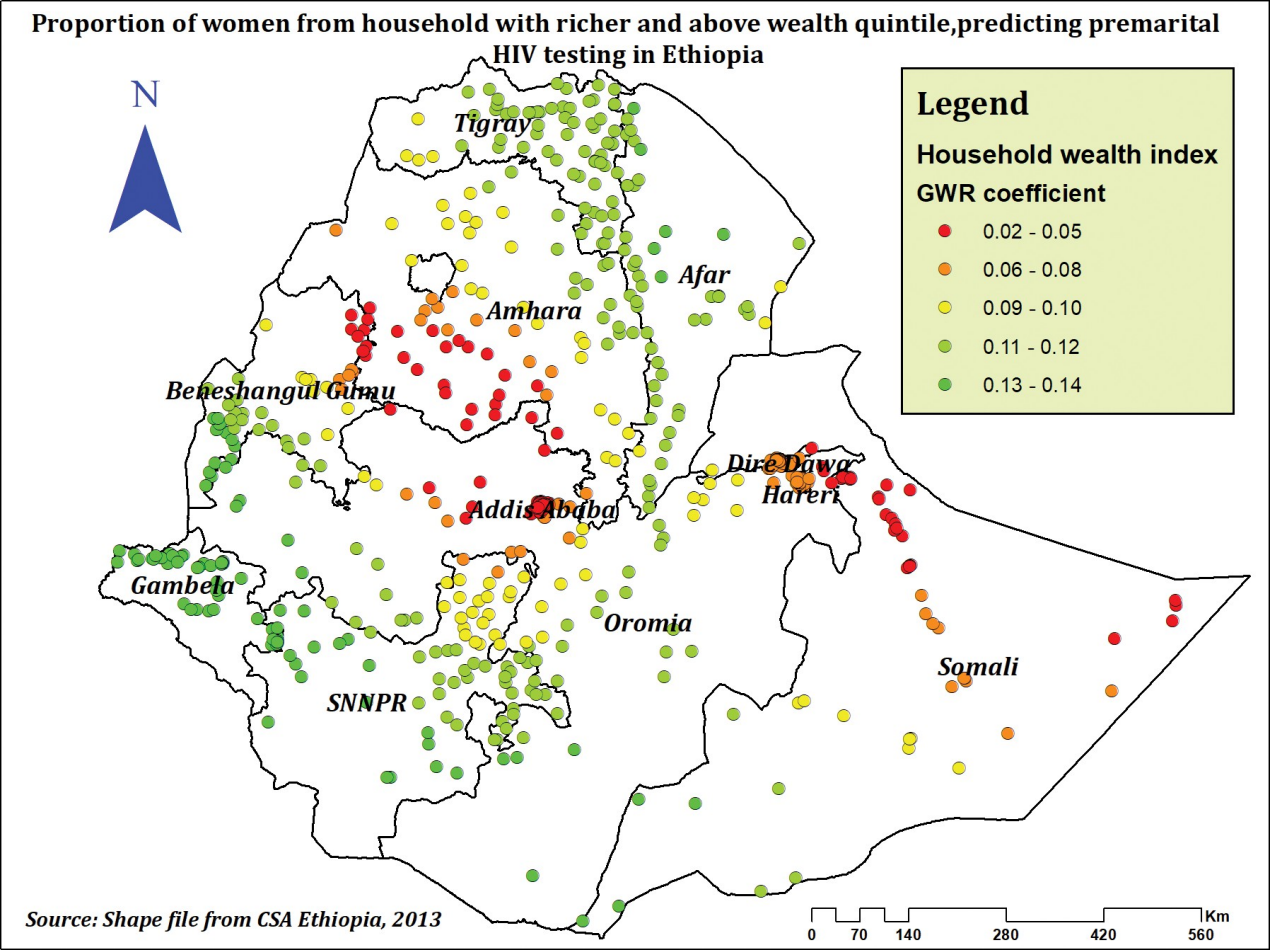
The GWR coefficients of households with richer and above wealth quintile predicting premarital HIV testing among married women in Ethiopia, using 2016 EDHS.

Premarital HIV testing prevalence and the proportion of women from richer and higher quintile households were found to be positively related. As the percentage of women living in households with wealth in the top quintile rose, premarital HIV testing prevalence was found to be highest in Tigray, Afar, Gambela, various SNNPR regions, west and southern Oromia, and west and southern Benishangul (**Fig 9**).

**Fig 10:**
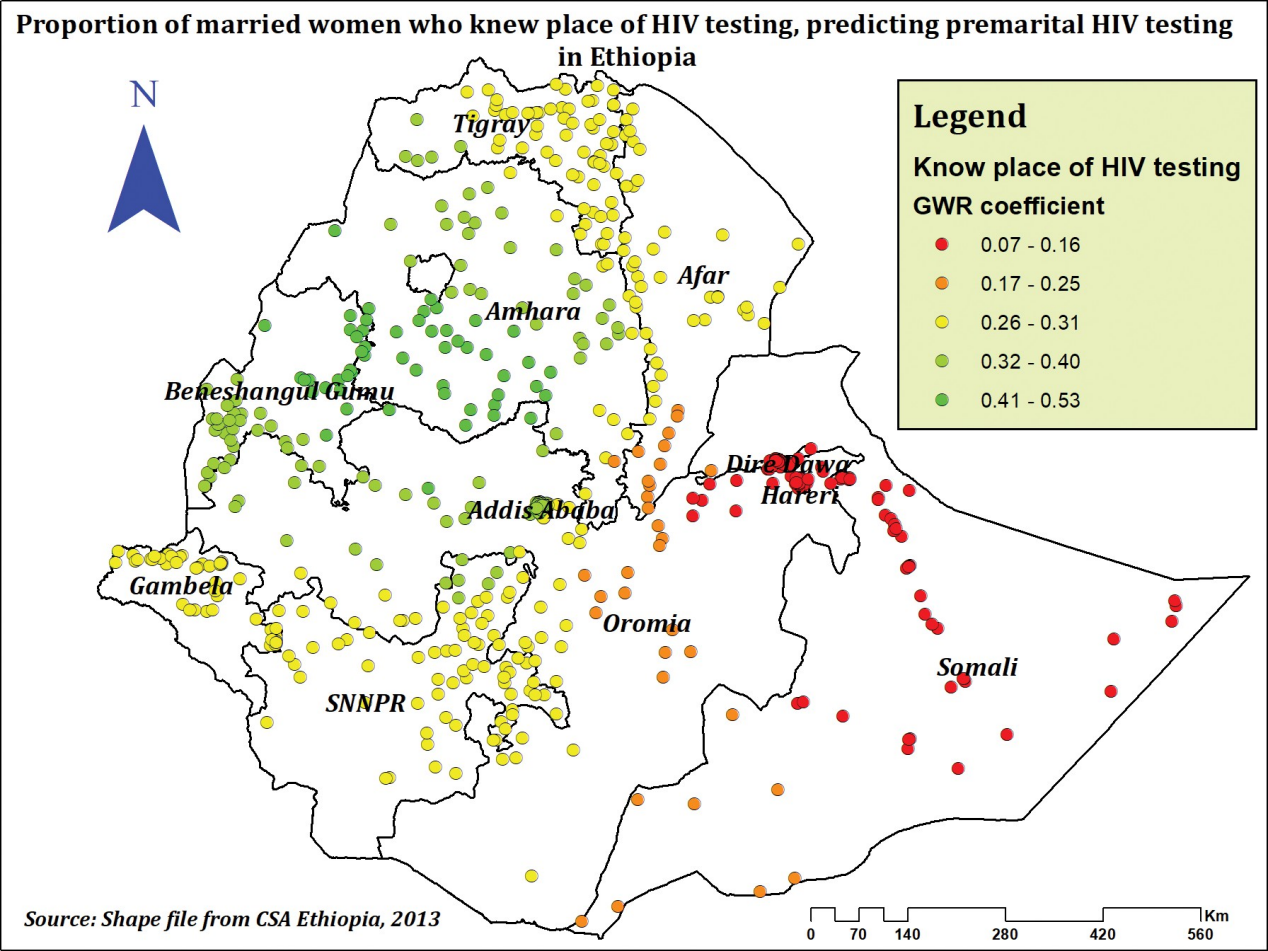
The GWR coefficients of women who know place of HIV testing predicting premarital HIV testing among married women in Ethiopia, using 2016 EDHS.

The percentage of women who were aware of where to get tested for HIV was positively correlated with the frequency of premarital HIV testing. Premarital HIV testing prevalence increased at the highest rates in Addis Ababa, Amhara, Benishangul Gumuz, and western Tigray as the percentage of women who knew where to get tested for HIV rose (**Fig 10**).

**Fig 11:**
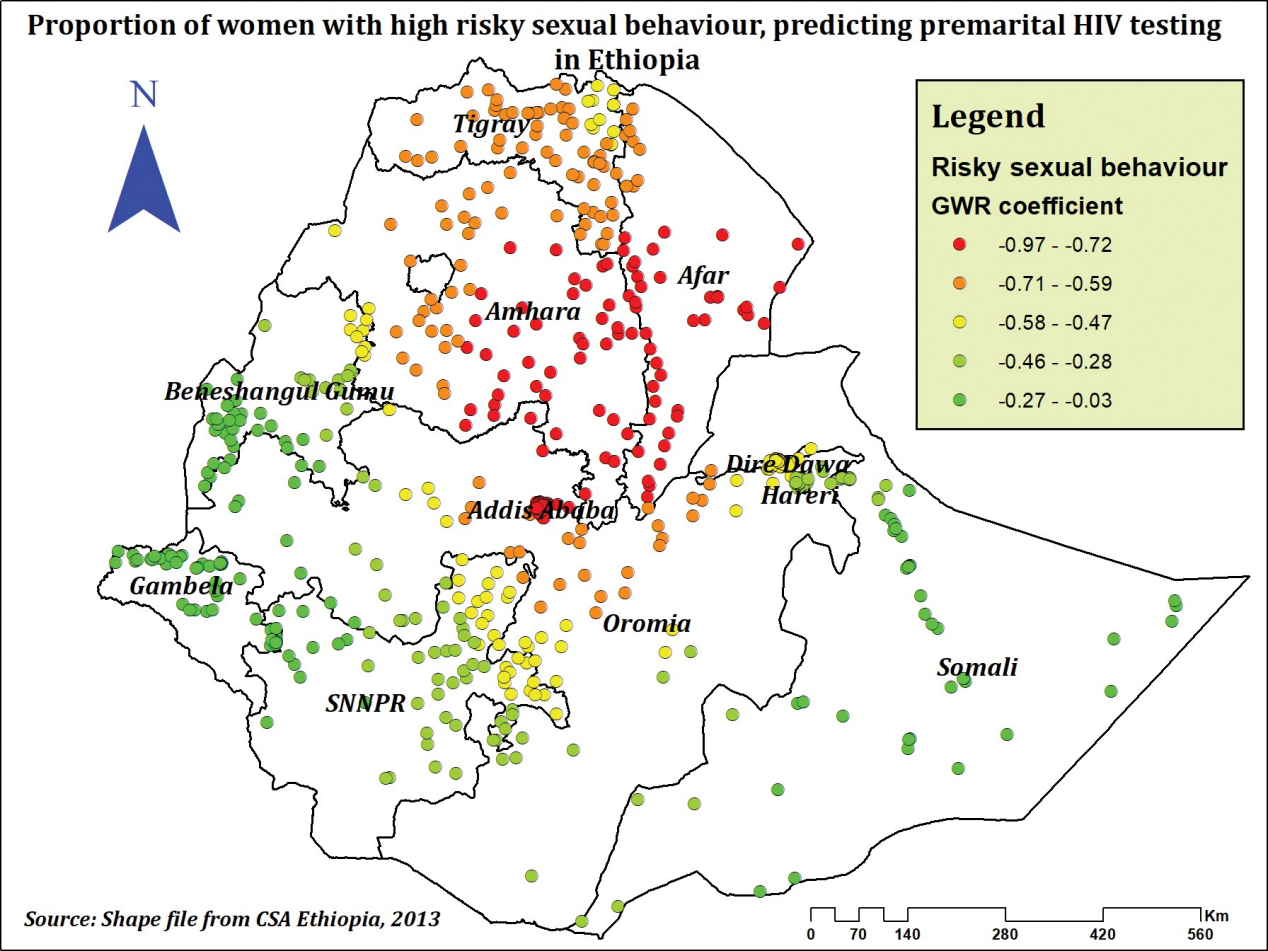
The GWR coefficients of high risky sexual behavior predicting premarital HIV testing among married women in Ethiopia, using 2016 EDHS.

High-risk sexual behavior and the prevalence of premarital HIV testing had a negative correlation. The prevalence of premarital HIV testing significantly reduced in Gambela, Benishangul Gumuz, Somalia, Hareri, Dire Dawa, the majority of SNNPR, and some areas of Oromia as the proportion of women engaging in high-risk sexual behaviors rose (**Fig 11**).

**Fig 12:**
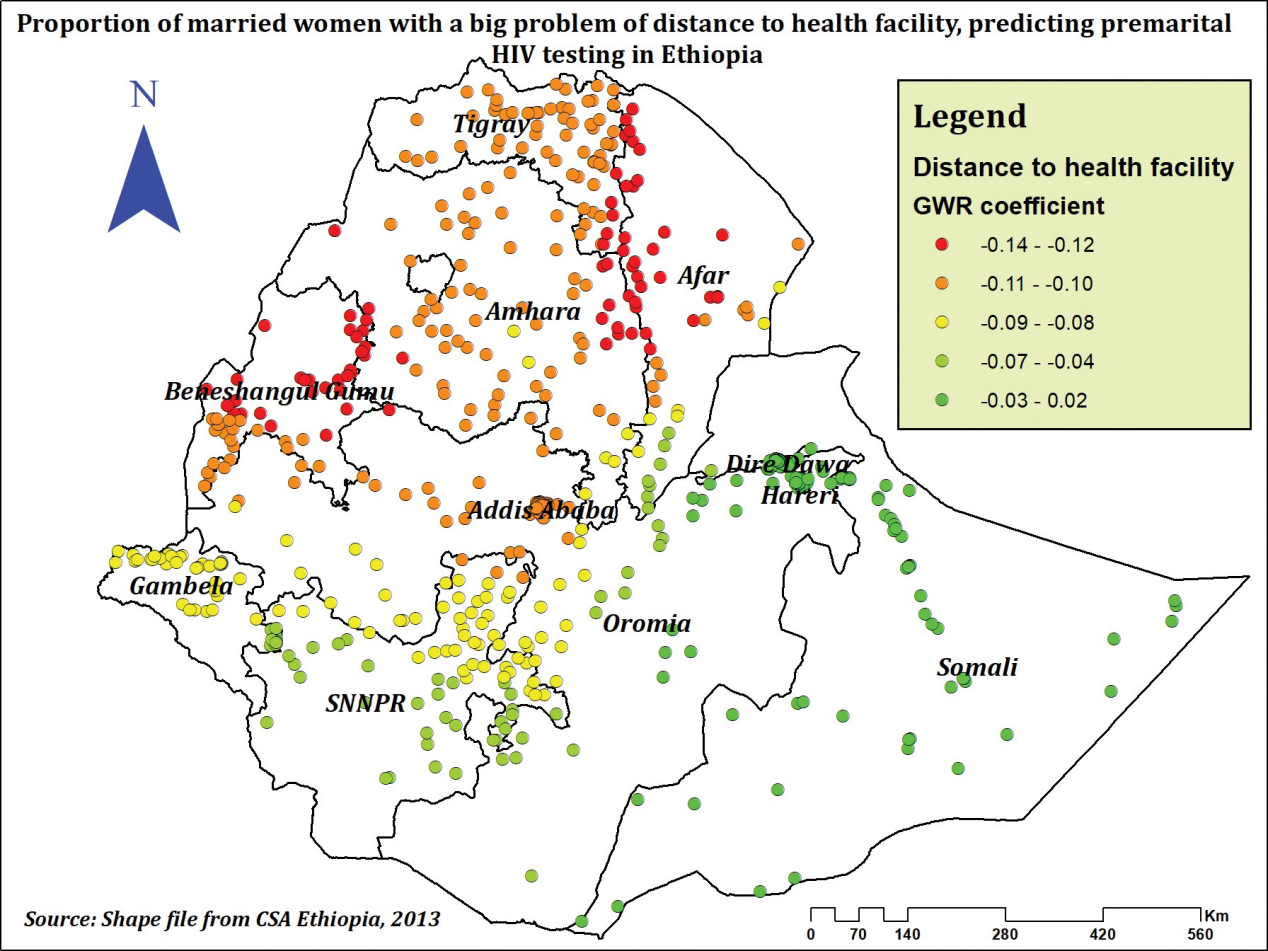
Estimated GWR coefficients for a big problem of distance to health facility predicting premarital HIV testing among married women in Ethiopia, using 2016 EDHS.

Distance to a health facility had a negative effect on the prevalence of premarital HIV testing. As the proportion of women who reported distance to health facilities as a big problem increased, the prevalence of premarital HIV testing was highly decreased at Dire Dawa, Hareri, Somali, some parts of Oromia, some parts of Afar, and some parts of SNNPR (**Fig 12**).

## Discussion

In order to comprehend the regional variation as well as the factors influencing the observed regional variation, this study examined the geographical variation of premarital HIV testing and its associated factors among married reproductive-age women across regions of Ethiopia.

In a spatial regression analysis, factors that significantly explained the spatial variation in premarital HIV testing included secondary and above education level, richer and above household wealth quintile, household media exposure, a problem with distance to a health facility, engaging in high-risk sexual behavior, and knowing the place of the HIV testing facilities. So, for these important variables, we mapped the local coefficients. In the multilevel mixed-effect robust poisson regression analysis, premarital HIV testing was positively related with education level, household media exposure, wealth index, khat chewing, prior history of HIV testing, knowledge of HIV, distance to a health facilities, and community-level media exposure. While, premarital HIV testing was negatively correlated with women’s age, religion, HIV-related stigma, and age at first sex.

Only 24.5% [95% CI: 23.65–25.32%] of women who were married or living with partner during data collection had undergone premarital HIV testing. This finding is lower than previous report from Ethiopia [54], Nigeria [55], South Africa [56], Ghana [57] and China [58]. However, our finding higher than previous reports from Nigeria [59, 60]. This discrepancy might be due to population and time difference across studies. Premarital HIV testing was spatially clustered at the cluster level, and Getis-Ord spatial analysis revealed a significant hot and cold spots. Geographical variation in HIV testing has been reported by numerous studies conducted around the world [30, 57, 61, 62]. This could be due to participant cultural, behavioral, and lifestyle variations among areas. In addition, the observed variation in HIV prevalence across these communities may be reflected in premarital HIV testing patterns.

According to the results from spatial regression models, a positive relationship between percentage of women with secondary and higher education levels and premarital HIV testing hotspots was revealed. The multilevel analysis also supports that educated women had a higher prevalence of premarital HIV testing compared to those who had not attended formal education. This finding is consistent with previous findings from Ethiopia [25, 27, 32], Nepal [29], Nigeria [22], and a study conducted among women from east African countries [24]. This might be brought on by differences in educational opportunity and quality across regions [63] and when education levels increase, knowledge and attitudes towards HIV and HIV testing also increase [64]. Education empowers women to make decisions related to their health, control over resources, and voice, and helps women get employment, which makes them financially independent and economically strong [65].

In both spatial and multilevel regressions, premarital HIV testing was positively related with household media exposure. Women from households with media exposure to any form of media, such as TV, radio, and newspapers, have a higher prevalence of premarital HIV testing compared to their counterparts. Similar results were reported from, Ghana [66], Nepal [29] and Papua New Guinea [67]. Media exposure has a strong connection to household wealth and expanding access to communication technology [68], which might positively enhance awareness of HIV testing and prevention [69].

Knowledge of the place of HIV testing showed a positive association with the hotspot areas for premarital HIV testing. And also women who had a previous history of HIV testing showed a higher prevalence of premarital HIV testing compared to their counterparts. which is congruent with a study conducted in Ethiopia [31]. This might be due to the good impact that knowing where to get tested for HIV has on having a thorough understanding of HIV/AIDS [70] and an increase in knowledge and attitude towards HIV after first-time exposure to HIV testing. This could be the reason that women with comprehensive HIV-related knowledge in our study also showed a higher prevalence of premarital HIV testing compared to women who had low HIV-related knowledge. Which is supported by other studies from Ethiopia [30], Nigeria [22], and a study conducted among east African women [24].

Women from households with the richest wealth quintile had an increased prevalence of premarital HIV testing compared to those from the poorest wealth quintile. The spatial result also revealed premarital HIV testing hot spots had a positive relationship with being from a richer or higher wealth quintile, consistent with studies conducted in Ethiopia [30, 71], Ghana [57], Gambia [20], and Nigeria [22]. A possible explanation for our finding might be that individuals, especially women from the lower wealth quintile, may have low HIV-related and mother-to-child transmission knowledge [72]. In addition, low household economic status may negatively affect women’s health interventions and utilization [73]. Thus, socio-economically disadvantaged women may have a lower chance of HIV testing [74]. However, our finding is different from a study conducted among East African women [24] and Nepal [29]. This might be due to population and living standard differences between populations. Since the previous study included women from different countries and found differences in economic status and quality of life across the countries, this may contribute [75].

Having high-risk sexual behavior was negatively related to hot spot areas of premarital HIV testing among married women. The reason might be fear of stigma and discrimination [76, 77]. This could be the reason why women who had a high perceived level of HIV-related stigma had a decreased prevalence of premarital HIV testing compared to those who had no perceived HIV-related stigma. Other studies from Ethiopia [21, 30, 78] and Nigeria [22] validated the evidence, which can be explained by perceived internalized HIV-related stigma, which may cause fear of disclosure, a feeling of shame and isolation, and despair. Thus, they may keep themselves from utilizing HIV testing services [79]. In contrast to our findings, a study among East African women [24] revealed a positive association between high-risk sexual behavior and premarital HIV testing, which may be accounted for by the fact that the study’s largest negative effect was frequently observed in undeveloped regions with mostly pastoral lifestyles, where health indicators and general wellbeing are low [63], and differences in culture, education, and HIV testing and counseling programs implemented across East African countries may also contribute.

Compared to women in the 15–19 age group, the prevalence of premarital HIV testing was lower among women in the age groups of 20–24, 25–29, 30–34, 35–39, 40–44, and 45–49. This finding is different from those of studies carried out in Nigeria [22] and among women in east Africa [24]. A possible justification could be that getting tested for HIV depends on a variety of factors, thus getting tested just because of getting older is not a guarantee. It may be advantageous to increase the uptake of HIV testing by using various, properly developed programs and methods that can deal with societal values and culture while addressing HIV risk perception and accurate information about HIV testing [80].

In comparison to women who had never chewed khat, those who had a history of doing so had a higher prevalence of premarital HIV testing. This could be as a result of how chewing khat affects cognitive processes like memory, learning, and response inhibition [81]. As a result, khat users may experience risky sexual activity at a young age [82]. They may therefore request HIV testing in order to learn their status, reduce their stress, and stop worrying.

In comparison to women who had their first sexual experience after turning 20, those who had their first sexual contact before turning 20had a lower prevalence of premarital HIV testing. This finding is in contrast to another study [24]. This might be a population difference taken into account in the studies, which is why the prior study included women from East Africa given that sociocultural practices and HIV preventive methods vary across nations [83].

Women with no problem with distance to a health facility had a higher prevalence of premarital HIV testing compared to those who reported the problem. The spatial analysis also revealed hotspot areas of premarital HIV testing were negatively associated with having a significant problem with distance to healthcare facilities. Previous findings from Ethiopia [71] and Malawi [84] also support this evidence. This might be a result of how long travel times to medical facilities affect outreach initiatives, health education initiatives, and the availability of HIV testing services [28].

Compared to women who were living in the Tigray region, women who were living in the Amhara region had an increased prevalence of premarital HIV testing. While women who were living in Oromia, Somali, Benishangul, Hareri, and Dire Dawa regions had a lower prevalence of premarital HIV testing compared to women who were living in the Tigray region, this regional difference may be explained by variations in HIV prevention strategies, health care financing, and the implementation of various strategic plans in across regions [85]. Women from clusters with high levels of media exposure had a higher prevalence of premarital HIV testing. This finding is supported by evidence from Kenya [86], Ghana [87], and sub-Saharan Africa [88]. Women who have been exposed to the media may be more likely than other women to receive HIV tests [89] due to the positive effect of media exposure on HIV-related knowledge and attitudes [90].

Our findings suggest that considering combined spatial and statistical analyses for examining the regional disparity of premarital HIV testing and the spatial factors responsible for the geographical discrepancy may work together and aid in pinpointing areas with low and high rates of premarital HIV testing and the development of context- and area-based interventions.

## Strength and limitations of the study

A validated data collection tool was used to obtain nationally representative data used for this study. Premarital HIV testing prevalence was taken into account in our study, and factors associated with the desired outcome of interest at the national level were found using robust Poisson regression. In addition, we employed a spatial analysis to pinpoint hotspot and coldspot areas of premarital HIV testing and identify spatial explanatory variables for this significant spatial variation in a specific geographic area. The drawbacks of a cross-sectional study design, however, apply to our work as well. As a result, we were unable to prove that the explanatory variables and outcome were causally related. In order to maintain respondent confidentiality, including individuals who have not had HIV testing, the GPS position is randomly shifted. Thus, positional errors of 0–2 kilometers in urban clusters, 0–5 kilometers in rural clusters, and 0–10 kilometers in 1% of rural clusters is included in the study. This might have an impact on local estimates and make it challenging to determine where the instances actually are.

## Conclusion

Premarital HIV testing is still low and notable regional differences across regions of Ethiopia were observed. In Addis Ababa, Dire Dawa, North Tigray, and several areas of the Afar and Amhara regions, it was shown to be statistically and significantly clustered. Number individual and community-level socio-demographic, socio-economic, behavioral and structural factors combined to cause a difference in risk and spatial variation of premarital HIV testing. As a result, in cold-spot areas, area-based prevention and interventional measures are necessary to reduce the contribution of heterosexual transmission to the HIV burden. Regional health care delivery services would also be more cost-effective if geographical explanatory factors were considered rather than just providing services at random.

## Supporting information

S1 Fig Flow chart showing sampling for weighted sample included in the study. (TIF)

## References

[1] World Health Organization. THE GLOBAL HEALTH OBSERVATORY(HIV). 2022 July 2022 [cited 2023; Available from: https://www.who.int/data/gho/data/themes/hiv-aids.

[2] Kharsany A.B. and Karim Q.A., HIV infection and AIDS in sub-Saharan Africa: current status, challenges and opportunities. The open AIDS journal, 2016. 10: p. 34. doi: 10.2174/1874613601610010034 27347270 PMC4893541

[3] UNAIDS. Global HIV & AIDS statistics—Fact sheet. 2023; Available from: https://www.unaids.org/en/resources/fact-sheet.

[4] world Bank. Prevalence of HIV, total (% of population ages 15–49)—Ethiopia. 2023; Available from: https://data.worldbank.org/indicator/SH.DYN.AIDS.ZS?contextual=min&locations=ET.

[5] Deribew A., et al., The Burden of HIV/AIDS in Ethiopia from 1990 to 2016: Evidence from the Global Burden of Diseases 2016 Study. Ethiop J Health Sci, 2019. 29(1): p. 859–868. doi: 10.4314/ejhs.v29i1.7 30700953 PMC6341438

[6] Dunkle K.L., et al., New heterosexually transmitted HIV infections in married or cohabiting couples in urban Zambia and Rwanda: an analysis of survey and clinical data. The Lancet, 2008. 371(9631): p. 2183–2191. doi: 10.1016/S0140-6736(08)60953-8 18586173

[7] Nnaji G., et al., Premarital HIV testing on prospective couples in a teaching hospital in sub Saharan Africa. Nigerian Journal of Medicine, 2014. 23(1): p. 13–19. 24946449

[8] Glynn J.R., et al., HIV risk in relation to marriage in areas with high prevalence of HIV infection. J Acquir Immune Defic Syndr, 2003. 33(4): p. 526–35. doi: 10.1097/00126334-200308010-00015 12869843

[9] World Health Organization. HIV. 2022 [cited 2023 January 5]; Available from: https://www.who.int/news-room/fact-sheets/detail/hiv-aids.

[10] Fonner V.A., et al., Voluntary counseling and testing (VCT) for changing HIV-related risk behavior in developing countries. Cochrane Database Syst Rev, 2012. 9(9): p. Cd001224. doi: 10.1002/14651858.CD001224.pub4 22972050 PMC3931252

[11] Federal Minstry of Health (Ethiopia). Guidelines for HIV Counselling and Testing in Ethiopia 2007 [cited 2023 January 5]; Available from: https://www.ilo.org/wcmsp5/groups/public/—ed_protect/—protrav/—ilo_aids/documents/legaldocument/wcms_125384.pdf.

[12] Pandve H.T., Premarital testing for HIV infection: Marriage bureaus should lead. Indian Journal of Dermatology, Venereology and Leprology, 2008. 74: p. 215. 18583785 10.4103/0378-6323.41365

[13] Kelley A.L., et al., Promotion of couples’ voluntary HIV counseling and testing: a comparison of influence networks in Rwanda and Zambia. BMC Public Health, 2016. 16(1): p. 744. doi: 10.1186/s12889-016-3424-z 27502690 PMC4977827

[14] Ayatollahi J., et al., Acceptability of HIV/AIDS testing among pre-marital couples in Iran (2012). Niger Med J, 2014. 55(4): p. 294–8. doi: 10.4103/0300-1652.137188 25114363 PMC4124541

[15] Shisana O., et al., Does marital status matter in an HIV hyperendemic country? Findings from the 2012 South African National HIV Prevalence, Incidence and Behaviour Survey. AIDS Care, 2016. 28(2): p. 234–41. 10.1080/09540121.2015.1080790 PMC5146982 26551532

[16] Habte D., Deyessa N., and Davey G., Assessment of the utilization of pre-marital HIV testing services and shabbir ismael determinants of VCT in Addis Ababa, 2003. Ethiopian Journal of Health Development, 2006. 20(1): p. 18–23.

[17] Manakandan S.K. and Sutan R., Expanding the role of Pre-marital HIV screening: Way forward for zero New infection. Open Journal of Obstetrics and Gynecology, 2016. 7(1): p. 71–79.

[18] Branson B.M., et al., Revised recommendations for HIV testing of adults, adolescents, and pregnant women in health-care settings. Morbidity and Mortality Weekly Report: Recommendations and Reports, 2006. 55(14): p. 1–CE-4. 16988643

[19] Mabala R., From HIV prevention to HIV protection: addressing the vulnerability of girls and young women in urban areas. Environment and Urbanization, 2006. 18(2): p. 407–432.

[20] Deynu M., Agyemang K., and Anokye N., Factors Associated with HIV Testing among Reproductive Women Aged 15–49 Years in the Gambia: Analysis of the 2019–2020 Gambian Demographic and Health Survey. International Journal of Environmental Research and Public Health, 2022. 19(8): p. 4860. doi: 10.3390/ijerph19084860 35457730 PMC9031325

[21] Erena A.N., Shen G., and Lei P., Factors affecting HIV counselling and testing among Ethiopian women aged 15–49. BMC Infectious Diseases, 2019. 19(1): p. 1076. doi: 10.1186/s12879-019-4701-0 31864297 PMC6925845

[22] Lépine A., Terris-Prestholt F., and Vickerman P., Determinants of HIV testing among Nigerian couples: a multilevel modelling approach. Health Policy and Planning, 2014. 30(5): p. 579–592. doi: 10.1093/heapol/czu036 24906362

[23] Mahande M.J., Phimemon R.N., and Ramadhani H.O., Factors associated with changes in uptake of HIV testing among young women (aged 15–24) in Tanzania from 2003 to 2012. Infectious diseases of poverty, 2016. 5(05): p. 64–75. doi: 10.1186/s40249-016-0180-3 27595846 PMC5011841

[24] Worku M.G., Tesema G.A., and Teshale A.B., Prevalence and associated factors of HIV testing among reproductive-age women in eastern Africa: multilevel analysis of demographic and health surveys. BMC Public Health, 2021. 21(1): p. 1262. doi: 10.1186/s12889-021-11292-9 34187431 PMC8243417

[25] Ahmed M. and Seid A., Factors associated with premarital HIV testing among married women in Ethiopia. PLOS ONE, 2020. 15(8): p. e0235830. doi: 10.1371/journal.pone.0235830 32745083 PMC7398550

[26] Ahmed M., et al., Uptake of premarital HIV testing and associated factors among women who had autonomous versus non autonomous marriage in Ethiopia: A nationwide study. PLOS ONE, 2022. 17(8): p. e0271879. doi: 10.1371/journal.pone.0271879 35980877 PMC9387857

[27] Bekele Y.A. and Fekadu G.A., Factors associated with HIV testing among young females; further analysis of the 2016 Ethiopian demographic and health survey data. PLOS ONE, 2020. 15(2): p. e0228783. doi: 10.1371/journal.pone.0228783 32045460 PMC7012428

[28] Benyumiza D., et al., Factors Associated with Utilization of HIV Testing Services among Adolescents Aged 10–19 Years in Lira District, Northern Uganda: A Cross-Sectional Study. BioMed Research International, 2021. 2021: p. 9568148. doi: 10.1155/2021/9568148 34423039 PMC8376469

[29] Bhattarai N., et al., Factors associated with HIV testing and counselling services among women and men in Nepal: a cross-sectional study using data from a nationally representative survey. BMJ Open, 2021. 11(12): p. e049415. doi: 10.1136/bmjopen-2021-049415 34862281 PMC8647541

[30] Alem A.Z., Liyew A.M., and Guadie H.A., Spatial pattern and associated factors of HIV testing and counselling among youths (15–24 years) in Ethiopia. BMC Public Health, 2021. 21(1): p. 1–13. 33794831 10.1186/s12889-021-10677-0 PMC8017837

[31] Temam G. and Ali A., Prevalence of HIV and discordant rate and their associated factors among premarital Voluntary Counseling and Testing (VCT) clients in Addis Ababa public VCT centers, Addis Ababa, Ethiopia. Ethiopian Journal of Health Development, 2012. 26(3): p. 160–168.

[32] Dillnessa E. and Enquselassie F., Couples voluntary counselling and testing among VCT clients in Addis Ababa, Ethiopia. Ethiop Med J, 2010. 48(2): p. 95–103. 20608013

[33] Birhanu M.Y., et al., Married women pre-marital HIV testing status in Ethiopia: Individual and community level factor analysis. 2023. 10.3389/fmed.2023.913040 PMC10018750 36936216

[34] Wang M., Yan A., and Katz R., Researcher requests for inappropriate analysis and reporting: a U.S. national survey of consulting biostatisticians. Annals of internal medicine, 2018. In press. 10.7326/M18-1230 30304365

[35] Ntani G., et al., Consequences of ignoring clustering in linear regression. BMC Medical Research Methodology, 2021. 21(1): p. 139. doi: 10.1186/s12874-021-01333-7 34233609 PMC8265092

[36] Thommai J., Simulation Study on the Effects of Ignoring Clustering in Regression Analysis. 2019, University of Twente.

[37] Wang S.T., Yu M.L., and Lin L.Y., Consequences of analysing complex survey data using inappropriate analysis and software computing packages. Public Health, 1997. 111(4): p. 259–62. doi: 10.1038/sj.ph.1900359 9242041

[38] Mbotwa J., Singini I., and Mukaka M., Discrepancy between statistical analysis method and study design in medical research: Examples, implications, and potential solutions. Malawi Med J, 2017. 29(1): p. 63–65. doi: 10.4314/mmj.v29i1.14 28567201 PMC5442496

[39] Barros A.J. and Hirakata V.N., Alternatives for logistic regression in cross-sectional studies: an empirical comparison of models that directly estimate the prevalence ratio. BMC medical research methodology, 2003. 3(1): p. 1–13. doi: 10.1186/1471-2288-3-21 14567763 PMC521200

[40] Martinez B.A.F., et al., Odds Ratio or Prevalence Ratio? An Overview of Reported Statistical Methods and Appropriateness of Interpretations in Cross-sectional Studies with Dichotomous Outcomes in Veterinary Medicine. Frontiers in Veterinary Science, 2017. 4. doi: 10.3389/fvets.2017.00193 29177157 PMC5686058

[41] ICF, C.S.A.a.D.P. Demographic and Health Survey, 2016 (Ethiopia). 2017 [cited 2023 January 28]; Available from: https://dhsprogram.com/pubs/pdf/FR328/FR328.pdf.

[42] Torman V.B.L. and Camey S.A., Bayesian models as a unified approach to estimate relative risk (or prevalence ratio) in binary and polytomous outcomes. Emerging themes in epidemiology, 2015. 12(1): p. 1–10. 26097494 10.1186/s12982-015-0030-y PMC4473845

[43] Thompson M.L., Myers J., and Kriebel D., Prevalence odds ratio or prevalence ratio in the analysis of cross sectional data: what is to be done? Occupational and environmental medicine, 1998. 55(4): p. 272–277. doi: 10.1136/oem.55.4.272 9624282 PMC1757577

[44] Jackson M.C. and Waller L.A., Exploring Goodness-of-Fit and Spatial Correlation Using Components of Tango’s Index of Spatial Clustering. Geographical Analysis, 2005. 37(4): p. 371–382.

[45] Oden N., Adjusting Moran’s I for population density. Statistics in Medicine, 1995. 14(1): p. 17–26. doi: 10.1002/sim.4780140104 7701154

[46] Pandey M., Singh V., and Vaishya R., Geomatics approach for assessment of respiratory disease mapping. The International Archives of Photogrammetry, Remote Sensing and Spatial Information Sciences, 2014. 40(8): p. 205.

[47] Anselin L., Local indicators of spatial association—LISA. Geographical analysis, 1995. 27(2): p. 93–115.

[48] Cheng W. and Washington S.P., Experimental evaluation of hotspot identification methods. Accident Analysis & Prevention, 2005. 37(5): p. 870–881. doi: 10.1016/j.aap.2005.04.015 15963449

[49] Srinivasan S., Spatial Regression Models, in Encyclopedia of GIS, Shekhar S. and Xiong H., Editors. 2008, Springer US: Boston, MA. p. 1102–1105.

[50] Martin C. and Stewart F., Geographically Weighted Regression: A Tutorial on using GWR in ArcGIS 9.3. Maynooth, County Kildare, Ireland: National Centre for Geocomputation, National University of Ireland Maynooth, 2009: p. 1–27.

[51] ESRI press. ArcGIS Pro Tool Reference (How Geographically Weighted Regression (GWR) works). Available from: https://pro.arcgis.com/en/pro-app/latest/tool-reference/spatial-statistics/how-geographicallyweightedregression-works.htm.

[52] Burnham K.P., Model selection and multimodel inference. A practical information-theoretic approach, 1998.

[53] ESRI press. How OLS regression works. [cited March 10, 2023; Available from: https://pro.arcgis.com/en/pro-app/latest/tool-reference/spatial-statistics/how-ols-regression-works.htm.

[54] Abdissa D., Tazebew M., and Gerbi A., Prevalence of Voluntary Counseling and Testing Utilization and Its Associated Factors Among Merawi Preparatory School Students in Merawi Town, West Gojjam, Ethiopia. HIV AIDS (Auckl), 2020. 12: p. 923–930. 33363410 10.2147/HIV.S281955 PMC7754085

[55] Ogaji D., Oyeyemi A., and Ibrahim I., Awareness, willingness and use of Voluntary HIV testing and counseling services by students of a university in south-south Nigeria. Journal of Community Medicine and Primary Health Care, 2013. 25(2): p. 36–44.

[56] Kilembe W., et al., Implementation of couples’ voluntary HIV counseling and testing services in Durban, South Africa. BMC public health, 2015. 15: p. 601. doi: 10.1186/s12889-015-1959-z 26136116 PMC4489128

[57] Nutor J.J., et al., Geographical variations and factors associated with recent HIV testing prevalence in Ghana: spatial mapping and complex survey analyses of the 2014 demographic and health surveys. BMJ Open, 2021. 11(7): p. e045458. doi: 10.1136/bmjopen-2020-045458 34244255 PMC8273465

[58] Mao Y.-r., et al., [An epidemiological study on sexual transmission of human immunodeficiency virus among pre-marital group in Yining city, Xinjiang]. Zhonghua liu xing bing xue za zhi = Zhonghua liuxingbingxue zazhi, 2004. 25(4): p. 322–324. 15231201

[59] Oginni A., Obianwu O., and Adebajo S., Socio-demographic factors associated with uptake of HIV counseling and testing (HCT) among Nigerian youth. 2014.

[60] Ibrahim M., et al., Socio-demographic determinants of HIV counseling and testing uptake among young people in Nigeria. International Journal of Prevention and Treatment, 2013. 2(3): p. 23–31.

[61] Jooste S., et al., Geographical variation in HIV testing in South Africa: Evidence from the 2017 national household HIV survey. South Afr J HIV Med, 2021. 22(1): p. 1273. doi: 10.4102/sajhivmed.v22i1.1273 34522430 PMC8424727

[62] Tordoff D.M., et al., Geographic Variation in HIV Testing Among Transgender and Nonbinary Adults in the United States. JAIDS Journal of Acquired Immune Deficiency Syndromes, 2022. 89(5). doi: 10.1097/QAI.0000000000002909 35001041 PMC9058176

[63] Tesema M.T. and Braeken J., Regional inequalities and gender differences in academic achievement as a function of educational opportunities: Evidence from Ethiopia. International Journal of Educational Development, 2018. 60: p. 51–59.

[64] Gao X., et al., Effectiveness of school-based education on HIV/AIDS knowledge, attitude, and behavior among secondary school students in Wuhan, China. PLoS One, 2012. 7(9): p. e44881. doi: 10.1371/journal.pone.0044881 22970322 PMC3436789

[65] Habib K., et al., Impact of education and employment on women empowerment. European Online Journal of Natural and Social Sciences: Proceedings, 2019. 8(3 (s)): p. pp. 62–74.

[66] Manu A., et al., Risky sexual behaviours and HIV testing among young people in Ghana: evidence from the 2017/2018 Multiple Indicator Cluster Survey. Reprod Health, 2022. 19(1): p. 125. doi: 10.1186/s12978-022-01439-1 35643502 PMC9148450

[67] Adegboye O.A., et al., Media Exposure, Behavioural Risk Factors and HIV Testing among Women of Reproductive Age in Papua New Guinea: A Cross-Sectional Study. Trop Med Infect Dis, 2022. 7(2). 10.3390/tropicalmed7020030 PMC8875656 35202225

[68] Gashu K.D., et al., Factors associated with women’s exposure to mass media for Health Care Information in Ethiopia. A case-control study. Clinical Epidemiology and Global Health, 2021. 12: p. 100833.

[69] Asaduzzaman M., et al., Awareness and knowledge of HIV/AIDS among married women in rural Bangladesh and exposure to media: a secondary data analysis of the 2011 Bangladesh Demographic and Health Survey. Nagoya journal of medical science, 2016. 78(1): p. 109. 27019532 PMC4767519

[70] Agegnehu C.D., et al., Determinants of comprehensive knowledge of HIV/AIDS among reproductive age (15–49 years) women in Ethiopia: further analysis of 2016 Ethiopian demographic and health survey. AIDS Research and Therapy, 2020. 17(1): p. 51. 32787881 10.1186/s12981-020-00305-z PMC7425582

[71] Teklehaimanot H.D., et al., Factors influencing the uptake of voluntary HIV counseling and testing in rural Ethiopia: a cross sectional study. BMC Public Health, 2016. 16(1): p. 239. doi: 10.1186/s12889-016-2918-z 26955869 PMC4784416

[72] Faust L., Yaya S., and Ekholuenetale M., Wealth inequality as a predictor of HIV-related knowledge in Nigeria. BMJ Global Health, 2017. 2(4): p. e000461. doi: 10.1136/bmjgh-2017-000461 29333285 PMC5759704

[73] Benova L., et al., Socio-economic factors associated with maternal health-seeking behaviours among women from poor households in rural Egypt. International Journal for Equity in Health, 2014. 13(1): p. 111. doi: 10.1186/s12939-014-0111-5 25424200 PMC4247707

[74] Ekholuenetale M., Nzoputam C.I., and Okonji O.C., Association between socio-economic factors and HIV self-testing knowledge amongst South African women. South Afr J HIV Med, 2022. 23(1): p. 1347. doi: 10.4102/sajhivmed.v23i1.1347 35399747 PMC8991179

[75] My Life Elsewhere. QUALITY OF LIFE COMPARISON. [cited March 4 2023; Available from: https://www.mylifeelsewhere.com/compare/ethiopia/nepal#:~:text=Economy&text=Ethiopia%20has%20a%20GDP%20per,is%20%243%2C800%20as%20of%202020.&text=In%20Ethiopia%2C%2017.5%25%20of%20adults,is%203.0%25%20as%20of%202017.

[76] Blondell S., et al., Barriers and Facilitators to HIV Testing in Migrants in High-Income Countries: A Systematic Review. AIDS and behavior, 2015. 19. doi: 10.1007/s10461-015-1095-x 26025193

[77] Ahmed S.I., et al., Attitudes and barriers towards HIV screening: A qualitative study of people living with HIV/AIDS (PLWHA) in Malaysia. J Infect Prev, 2017. 18(5): p. 242–247. doi: 10.1177/1757177416689723 29317901 PMC5753937

[78] Sisay S., et al., Perception of High School Students on risk for acquiring HIV and utilization of Voluntary Counseling and Testing (VCT) service for HIV in Debre-berhan Town, Ethiopia: a quantitative cross-sectional study. BMC research notes, 2014. 7: p. 1–10. 25112147 10.1186/1756-0500-7-518 PMC4266969

[79] Center for Disease Control and Prevention. HIV STIGMA AND DISCRIMINATION. 2021 [cited March 4 2023; Available from: https://www.cdc.gov/hiv/basics/hiv-stigma/index.html#:~:text=HIV%20internalized%20stigma%20can%20lead,tested%20and%20treated%20for%20HIV.

[80] Musumari P.M., et al., Factors associated with HIV testing and intention to test for HIV among the general population of Nonthaburi Province, Thailand. PLoS One, 2020. 15(8): p. e0237393. doi: 10.1371/journal.pone.0237393 32797048 PMC7428091

[81] Ahmed A., et al., Khat and neurobehavioral functions: A systematic review. PLoS one, 2021. 16(6): p. e0252900. doi: 10.1371/journal.pone.0252900 34111184 PMC8192015

[82] Berhanu D., et al., Khat use among HIV voluntary counselling and testing centre clients in Ethiopia. Culture, health & sexuality, 2012. 14(10): p. 1197–1212. doi: 10.1080/13691058.2012.722684 22988913

[83] Virkud A.V., et al., Access to HIV prevention services in East African cross-border areas: a 2016–2017 cross-sectional bio-behavioural study. J Int AIDS Soc, 2020. 23 Suppl 3(Suppl 3): p. e25523. doi: 10.1002/jia2.25523 32602638 PMC7325514

[84] Palk L., et al., Travel time to health-care facilities, mode of transportation, and HIV elimination in Malawi: a geospatial modelling analysis. The Lancet Global Health, 2020. 8(12): p. e1555–e1564. doi: 10.1016/S2214-109X(20)30351-X 33220218 PMC7738178

[85] HEALTH M.O. HIV/AIDS National Strategic Plan for Ethiopia. 2022 [cited March 5 2023; Available from: https://www.prepwatch.org/wp-content/uploads/2022/07/Ethiopia-HIVAIDS-National-Strategic-Plan-2021-25.pdf.

[86] Onsomu E.O., et al., Importance of the Media in Scaling-Up HIV Testing in Kenya. SAGE Open, 2013. 3(3): p. 2158244013497721.

[87] Sano Y., et al., Exploring the linkage between exposure to mass media and HIV testing among married women and men in Ghana. AIDS Care, 2016. 28(6): p. 684–8. doi: 10.1080/09540121.2015.1131970 26753839

[88] Somefun O.D., Wandera S.O., and Odimegwu C., Media exposure and HIV testing among youth in sub-Saharan Africa: evidence from demographic and health surveys (DHS). Sage Open, 2019. 9(2): p. 2158244019851551.

[89] Adamu Muhammad, H. and S. Godwin Matthew, Media Campaign Exposure and HIV/AIDS Prevention: 1980–2020, in AIDS Updates, O. Samuel Ikwaras, Editor. 2021, IntechOpen: Rijeka. p. Ch. 2.

[90] Muhammad Hamid A., Tamam E., and Nizam bin Osman M., Relationships between Media Exposure and Knowledge, Attitude, and Practice on HIV/AIDS: A Cross Sectional Survey of Adolescent Islamiyya Girls in Nigeria. Health Communication, 2020. 35(4): p. 419–429. doi: 10.1080/10410236.2018.1564960 30700145

