# Supplementary figures and images for: Spatial variation of premarital HIV testing and its associated factors among married women in Ethiopia: Multilevel and spatial analysis using 2016 demographic and health survey data
Source: PLoS One. 2023 Nov 30;18(11):e0293227. doi: 10.1371/journal.pone.0293227 (PMC10688645; doi:10.1371/journal.pone.0293227)

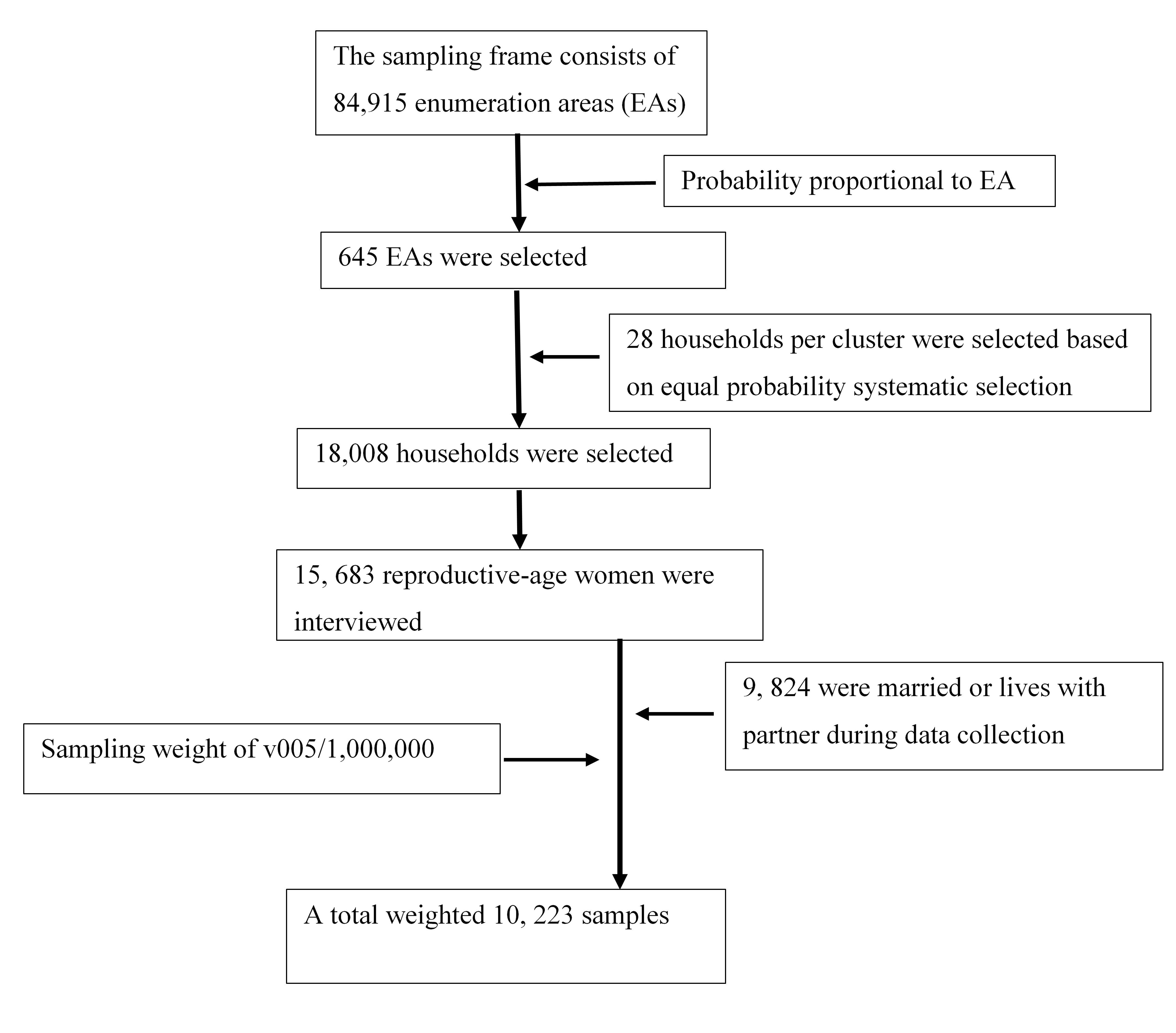

Supplement: S1 Fig — (TIF) [file pone.0293227.s001.tif]
